# A reference human genome dataset of the BGISEQ-500 sequencer

**DOI:** 10.1093/gigascience/gix024

**Published:** 2017-04-01

**Authors:** Jie Huang, Xinming Liang, Yuankai Xuan, Chunyu Geng, Yuxiang Li, Haorong Lu, Shoufang Qu, Xianglin Mei, Hongbo Chen, Ting Yu, Nan Sun, Junhua Rao, Jiahao Wang, Wenwei Zhang, Ying Chen, Sha Liao, Hui Jiang, Xin Liu, Zhaopeng Yang, Feng Mu, Shangxian Gao

**Affiliations:** 1 National Institutes for food and drug Control (NIFDC), No.2, Tiantan Xili Dongcheng District, Beijing 10050, P. R. China; 2 BGI-Shenzhen, Bei Shan Industrial Zone, Yantian District, Shenzhen, Guangdong Province, 518083, P. R. China; 3 State Food and Drug Administration Hubei Center for Medical Equipment Quality Supervision and Testing, 24-9, Zhongbei East Road, Wuhan, Hubei Province, 430000, P. R. China; 4 BGI-Qingdao, Tuanjie Rd., Huangdao District, Qingdao, Shandong Province, 266555, P. R. China

**Keywords:** genomics, sequencing, next-generation sequencing, BGISEQ-500

## Abstract

**Background:** BGISEQ-500 is a new desktop sequencer developed by BGI. Using DNA nanoball and combinational probe anchor synthesis developed from Complete Genomics™ sequencing technologies, it generates short reads at a large scale. **Findings:** Here, we present the first human whole-genome sequencing dataset of BGISEQ-500. The dataset was generated by sequencing the widely used cell line HG001 (NA12878) in two sequencing runs of paired-end 50 bp (PE50) and two sequencing runs of paired-end 100 bp (PE100). We also include examples of the raw images from the sequencer for reference. Finally, we identified variations using this dataset, estimated the accuracy of the variations, and compared to that of the variations identified from similar amounts of publicly available HiSeq2500 data. **Conclusions:** We found similar single nucleotide polymorphism (SNP) detection accuracy for the BGISEQ-500 PE100 data (false positive rate [FPR] = 0.00020%, sensitivity = 96.20%) compared to the PE150 HiSeq2500 data (FPR = 0.00017%, sensitivity = 96.60%) better SNP detection accuracy than the PE50 data (FPR = 0.0006%, sensitivity = 94.15%). But for insertions and deletions (indels), we found lower accuracy for BGISEQ-500 data (FPR = 0.00069% and 0.00067% for PE100 and PE50 respectively, sensitivity = 88.52% and 70.93%) than the HiSeq2500 data (FPR = 0.00032%, sensitivity = 96.28%). Our dataset can serve as the reference dataset, providing basic information not just for future development, but also for all research and applications based on the new sequencing platform.

## Background

Massively parallel sequencing technologies (also called as the second-generation sequencing) generate large amounts of data with lower cost, shorter reads, and higher single base error rate compared to Sanger sequencing technology [1]. With the large amount of data and well-developed analysis tools, second-generation sequencing data can be used to effectively and accurately identify genomic variations [2]. Thus it has been widely applied in both research and application [3]. Currently there are several commercially available second-generation sequencing platforms with differing performance and data features [4,5]. With more and more research areas and applications to apply sequencing to, new sequencing platforms are being developed at a rapid pace. The BGISEQ-500 sequencer was first announced by BGI in October 2015. It was developed based on the Complete Genomics™ sequencing technologies and applied DNA NanoBalls (DNBs) technology [6] for sequencing library construction and combined primer anchor synthesis (cPAS) for sequencing. We present here a dataset generated from the BGISEQ-500 sequencer, including examples of the raw images and the final sequences. We also conducted variation calling using this dataset and compared the variation calling result to that from other sequencers. This dataset can serve as a useful reference for the community to develop bioinformatics methods and sequencing-based applications on this new sequencing platform.

### DNA preparation

NA12878 cell line (Coriell Cat# GM12878, RRID:CVCL_7526) genomic DNA was ordered from the Coriell Institute, and it contained 50 μg per tube. The genomic DNA was quantified by Qubit 3.0 fluorometer (Life Technologies, Paisley, UK), and the integrity was qualified on the 2% agarose gel to make sure the genomic DNA molecular was larger than 23 kb and not substantially degraded.

### Sequencing library preparation

**Figure 1:**
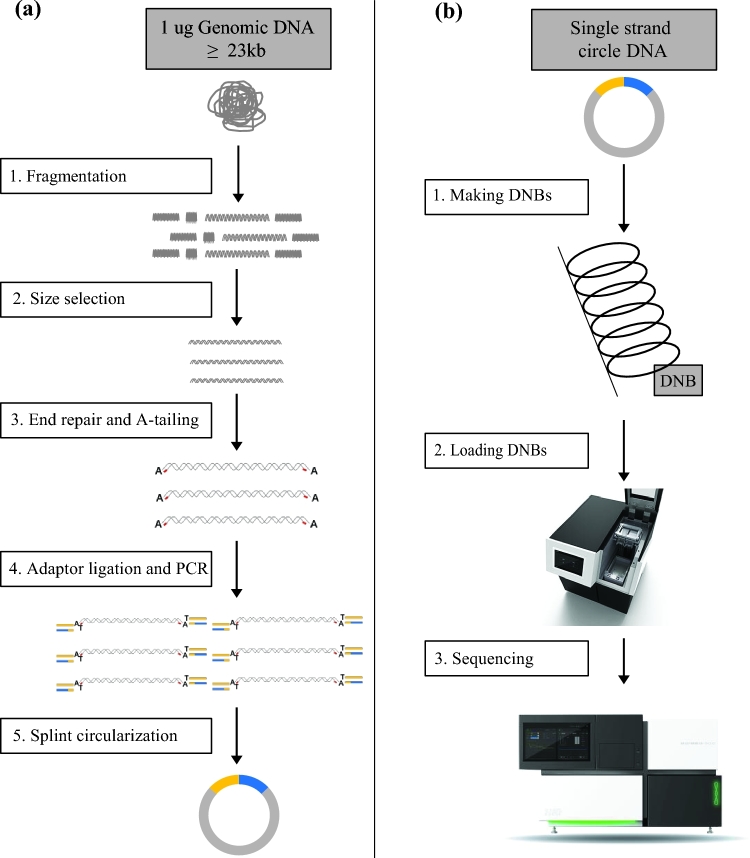
Flowchart of library construction and sequencing. The library construction includes fragmentation, size selection, end repair and A-tailing, adaptor ligation, PCR amplification, and splint circularization **(a)**. The sequencing includes making DNBs, loading DNBs and sequencing **(b)**.

For the sequencing library construction, the NA12878 genomic DNA was fragmented by ultrasound on Covaris E220 (Covaris, Brighton, UK) to DNA fragments between 50 bp and ∼800 bp according to the manufacturer's instructions. The fragmented DNA was further selected to between 100 bp and ∼300 bp by AMPure XP beads (AGENCOURT). The selected DNA fragments were then repaired to obtain a blunt end and modified at the 3’end to get a dATP as a sticky end. The dTTP tailed adapter sequence was ligated to both ends of the DNA fragments. The ligation product was then amplified for eight cycles and subjected to the following single-strand circularization process. The PCR product was heat-denatured together with a special molecule that was reverse-complemented to one special strand of the PCR product, and the single-strand molecule was ligated using DNA ligase. The remaining linear molecule was digested with the exonuclease, finally obtaining a single-strand circular DNA library (Fig. 1a).

### Sequencing

We conducted sequencing according to the BGISEQ-500 protocol (Fig. 1b). There were three steps, including making DNBs, loading DNBs, and sequencing. For making DNBs, a 6 ng single-strand circular DNA library was first PCR-amplified for 10 minutes in an 80 μl reaction volume with pure water, buffer, and DNB polymerase. After the PCR reaction, 20 μl DNBs stopping buffer was added to terminate the PCR reaction. Finally, we used the Qubit^®^ ssDNA Assay Kit to quantify the DNBs on a Qubit^®^ Fluorometer (concentration ≥10 ng/μL).

For loading DNBs, we first added 33 μl DNBs loading buffer to DNBs product from the last step, and the mixture was placed on the BGIDL-50 (the sample preparation machine). Then we selected the DNBs loading process (version: sample load 2.0) to load DNB onto the sequencing chip, which included 96 minutes’ loading time and 30 minutes’ incubation at room temperature.

Finally, for sequencing, we followed to the BGISEQ-500 protocol. We selected sequence control software version 1.1.0.10003, sequence process version 1.0.06, and Zebracall process version 0.5.0.13875 (the base calling software; a detailed description can be found in the next section) for sequencing. Sequencing was initiated after the sequencing reagents preloaded and sequencing chip was installed, and this process was finished in ∼72 hours.

### Base calling and raw images

During sequencing, four channels of 16-bit grayscale images were captured by high-resolution sCMOS with ∼5.5 million pixels per image. About 570K DNBs were loaded onto the grid-patterned arrays of spots, which were photolithographically etched and surface-modified on the sequencing chip. The spots were illuminated by the lasers with different wavelengths. Intensity from the neighboring channel would also be observed due to cross-talk effect. The sequences of DNBs were base called by the software Zebra call (base calling software developed for BGISEQ-500). After background subtraction and registration of images from four channels, intensities of DNBs were extracted according to a template of grid pattern. Correction within channels and neighbor cycles was applied to increase the quality and stabilization. The cross-talk between intensities from the four fluorophores is caused by the imperfect wavelength filtering of optical filters isolating the bands of wavelength from the four types of fluorophore molecules. A regression technique can identify correlations in our intensity data and correct for them. For example, in order to correct the cross-talk between two channels (C and G), the correction of the C background intensity was found by linear regression after eliminating DNBs that did have true signal in the C channel. Such DNBs were identified by searching for DNBs that had the C intensity as the maximum of the four intensities, and, to retain just DNBs that were not too dim or noisy, we took only DNBs that had less than 80% of the C intensity for the remaining three other intensities. This left us with reasonably well-performing DNBs that most likely did not contain C at the currently interrogated position. Linear regression was then carried out for these background G intensities as a function of the C intensities. All of the G intensities could in turn be corrected for this cross-talk from the C channel by subtracting from them the expected background intensity produced by a given C intensity. Such regression could be done for each channel, simultaneously correcting for all of the correlations outlined above using a multiple linear regression. After all correction steps, the base with the highest probability was called according to the scale of intensities. When the whole sequencing was finished, the binary file with bases and quality score were converted into FASTQ format with Phred+33 quality score.

In order to map the base call quality to Phred+33 score, a prior probability model was constructed by the scale of intensities from channels. Bases were separated into 10 404 groups according to different parameters that may affect the confidence level of base calling. The base calling error probabilities (*P*) of each group were calculated by the mismatch distribution from repeated sequencing of the standard reference genome. Quality scores (*Q*) were calculated by the definition of Phred+33 quality scores:

\[
Q = - 10 \log_{10} P
\]

A huge table was constructed and hard-coded into the base call program to look up a corresponding quality score by different parameters.

**Figure 2:**
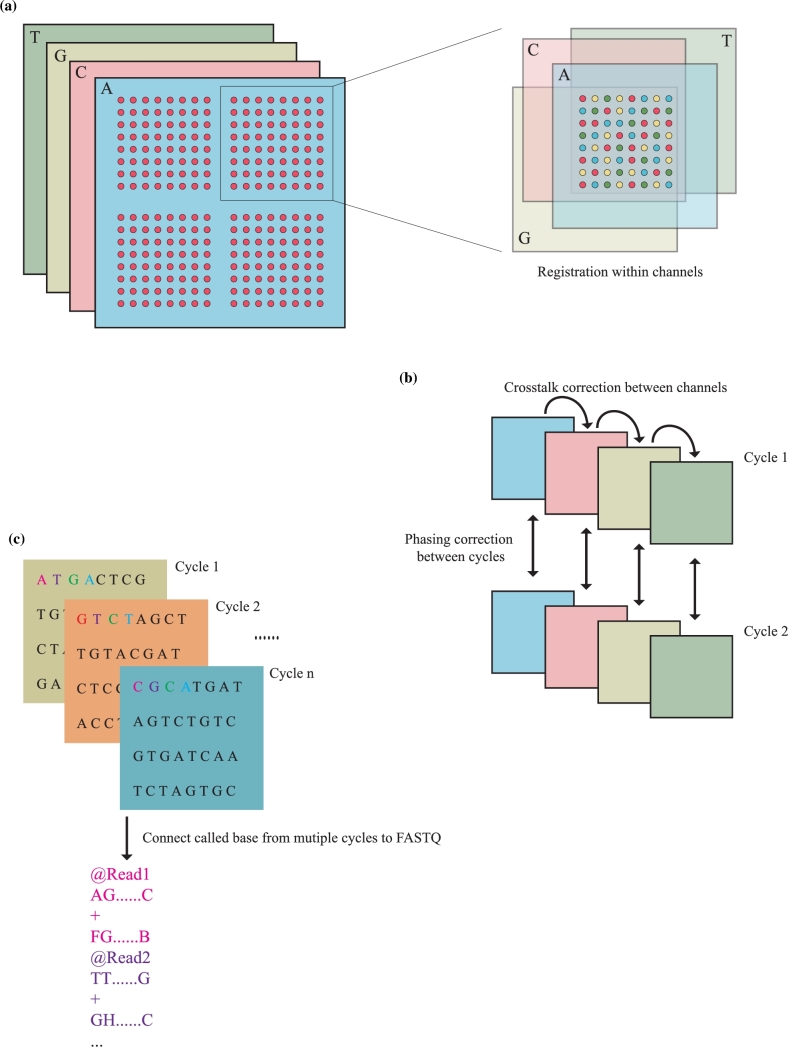
Raw image data processing on the BGISEQ-500 platform. **(a)** Registration of images from different channels. Relative coordinates will be calculated according to the pattern layout of DNBs. **(b)** Intensity correction between channels and cycles. Correction of the optical and chemical interferences on different channels and the neighbor cycles was applied. **(c)** Connecting called bases to FASTQ. Bases from all cycles will be collected and converted to FASTQ format. Phred score calculation and statistics will be applied during the conversion.

An example dataset of the images was included, and the base calling process was illustrated in Fig. 2.

## Results

### Sequencing data summary

**Figure 3:**
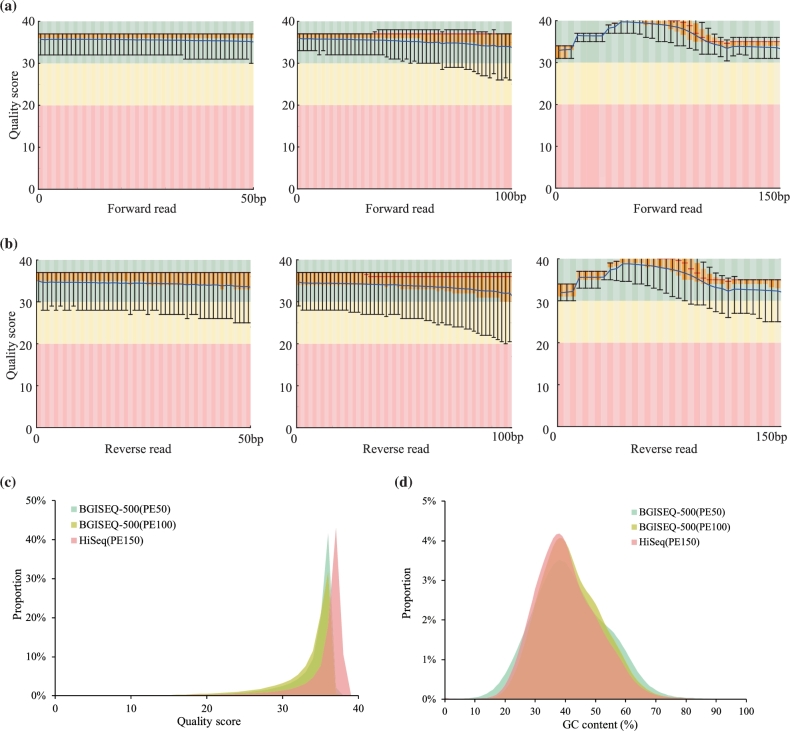
Quality control of the dataset after data filtering. Base-wise quality score distributions of the first read **(a)** from left to right (BGISEQ-500 PE50, BGISEQ-500 PE100, and HiSeq2500 PE150) and the second read **(b)** from left to right (BGISEQ-500 PE50, BGISEQ-500 PE100, and HiSeq2500 PE150). For each position along the reads, the quality scores of all reads were used to calculate the mean, median, and quantile values; thus the box plot can be shown. The overall quality score distribution of BGISEQ-500 and HiSeq2500 data **(c)**. GC content distribution of the BGISEQ-500 and HiSeq2500 data **(d)**. FastQC [18] was used for the calculation (FastQC, RRID:SCR_014583).

The sequencing data consists of four lanes, with two of PE100 and the other two of PE50 (Table 1). First, we analyzed the sequencing quality by identifying the low-quality reads. Although previous studies revealed that raw data filtering would not substantially affect variation calling result [7,8], we found slightly different performances of variation calling using different raw data filtering criteria (Table S1). Thus, we determined low-quality reads as reads that had more than 10% bases with sequencing quality lower than 10 and reads that had more than 1% Ns (ambiguous bases). In this way, we identified 11.9% (9.2% low-quality reads and 2.7% ambiguous reads) low-quality reads in PE100 data and 12.3% low-quality raw reads in PE50 data (5.4% low-quality reads and 6.9% ambiguous reads). In order to compare, we selected a similar amount of data (eight sequencing libraries and 16 lanes, PE150 reads, ∼98.5 Gbp data) from a public Illumina HiSeq2500 dataset of this cell line generated by Genome in a Bottle (GIAB) [9]. Using the same criteria for low-quality identification, we identified 7.95% low-quality reads (7.7% low-quality reads and 0.25% ambiguous reads). Excluding these low-quality reads, we then further analyzed the reads’ quality by plotting the distributions of base quality scores and GC content against those of the HiSeq2500 data (Fig. 3). Thus we found higher proportion of low-quality reads, more stable base quality distribution along the reads (Fig. 3a and b), and lower overall single base quality scores (Fig. 3c). And we observed some secondary peaks in the GC content distribution of BGISEQ-500 data, indicating higher GC bias (Fig. 3d).

**Table 1:** Summary of the dataset*

Sequencing Type	Read (×10^6^)	Bases (Gbp)	GC Content	>Q20	>Q30
PE50	2379	118.94	41.62%	96.00%	87.02%
PE100	1159	115.88	41.28%	96.39%	87.13%

*This dataset was from two runs of the BGISEQ-500 sequencer (PE50 and PE100). “>Q20/Q30 percentage” indicates the percent of bases with quality score (-10×lg(error rate)) higher than 20 and 30 (indicating error rates of 1% and 1‰, respectively).

### Variation calling and false positive/negative ratio estimation

**Figure 4:**
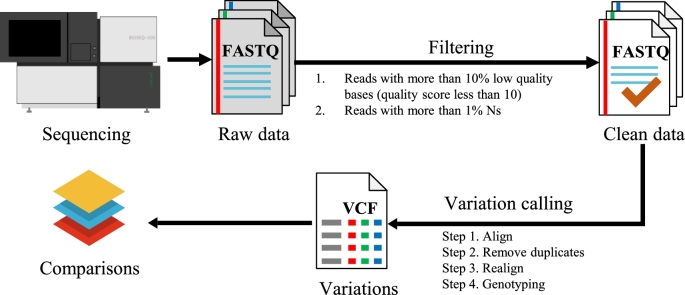
Variation calling based on the dataset. The major steps included data filtering, alignment, and variation calling, and the major parameters are also indicated.

In order to further depict the data quality and test applications of the new sequencing platform, we carried out variation calling using this dataset. We adapted the widely used pipeline (BWA [10] and GATK [11–13]; an illustration of the pipeline and key parameters can be found in Fig. 4a) for variation calling. We observed a higher mapping rate, similar sequencing coverage, and similar sequencing uniformity of the two BGISEQ-500 datasets compared to the HiSeq2500 dataset (Table 2). The lower unique mapping rate probably reflected the shorter read length of the dataset (2×50 bp and 2×100 bp compared to 2×150 bp). We also observed a slightly higher duplication rate and comparable mismatch rate in the BGISEQ-500 PE100 dataset compared to the HiSeq2500 data (Table 2).

**Table 2:** Mapping statistics of the dataset*

Metrics	BGISEQ-500 PE50	BGISEQ-500 PE100	HiSeq2500 PE150
Clean reads	2 378 725 921	1 136 008 901	708 941 148
Clean bases (bp)	118 936 296 050	113 600 890 100	104 923 289 904
Mapping rate	97.87%	99.22%	99.05%
Unique rate	93.17%	96.47%	97.06%
Duplicate rate	6.26%	2.47%	1.52%
Mismatch rate	0.34%	0.58%	0.56%
Average sequencing depth	37.57	37.44	34.52
Coverage	99.28%	99.12%	99.06%
Coverage at least 4×	98.90%	98.69%	98.60%
Coverage at least 10×	97.97%	97.81%	97.83%
Coverage at least 20×	95.78%	96.06%	94.81%

*The statistics shown here are calculated based on the clean reads (raw reads after filtering; the two platforms’ data went through the same filtering process). Unique mapping rate indicates the proportion of reads with unique alignment in the genome.

In total, we identified ∼3.4 million SNPs using the BGISEQ-500 datasets (3.45 million for PE50 data and 3.48 million for PE100 data); more than 3.6 million SNPs were identified using HiSeq2500 data (Table 3). For indels (insertion and deletions), we identified 842 058 from BGISEQ-500 PE100 data, compared to 553 842 identified from BGISEQ-500 PE50 data. Using the HiSeq2500 data, we identified 733 797 indels. The SNPs identified using BGISEQ-500 datasets were similar to those identified from HiSeq2500 data in different features including the dbSNP rate, proportion of SNPs in different regions related to genes, and transition/transversion (Ti/Tv) ratio, which indirectly reflected SNP accuracy. We also observed a similar situation for indels.

**Table 3:** Variation statistics of the dataset*

	BGISEQ-500 PE50	BGISEQ-500 PE100	HiSeq2500 PE150
SNPs	3 451 124	3 477 642	3 609 606
1000 genome and dbSNP	3 242 083	3 288 653	3 347 441
1000 genome specific	1260	420	693
dbSNP specific	180 935	179 967	243 256
dbSNP rate	99.19%	99.74%	99.48%
Novel	2846	8602	18 216
Homozygous	1 426 328	1 433 490	1 472 063
Heterzygous	2 024 796	2 044 152	2 137 543
Synonymous	19 880	20 012	20 860
Ti/Tv	2.0462	2.065	2.0427
dbSNP Ti/Tv	2.0608	2.0693	2.0503
Novel Ti/Tv	0.8948	0.9775	1.0544
Indels	553 842	842 058	733 797
1000 genome and dbSNP	260 157	320 741	314 161
1000 genome specific	7007	22 919	20 049
dbSNP specific	211 846	326 984	285 834
dbSNP rate	85.22%	76.92%	81.77%
Novel	74 832	171 414	113 753
Homozygous	206 163	295 492	300 013
Heterzygous	347 679	546 566	433 784

*1000 genome and dbSNP equals the number of SNPs that are found in both the 1000 genome and dbSNP databases (version 147 was used); 1000 genome specific equals the number of SNPs that are only found in the 1000 genomes database. dbSNP rate equals the number of SNPs found in the dbSNP database/total detected SNPs. Novel SNP equals the number of SNPs that are not found in the SNP database. Ti/Tv equals the ratio of SNP types that are transitional (/SNP type, transversional).

Further, to assess the accuracy of the variations, we used the high confident variations previously identified in NA12878 provided by GIAB [14]. Using the methods provided by GIAB, we estimated the false positive rates and sensitivity for BGISEQ-500 PE50 and PE100 data compared to those of HiSeq2500 data (Table 4). The SNP sensitivity was lower for the BGISEQ-500 datasets (96.20% for PE100 and 94.15% for PE50) than HiSeq2500 data (96.60%). And the SNP false positive rate (FPR) was similar for the BGISEQ-500 PE100 data (0.00020%) compared to HiSeq2500 data (0.00017%), and lower than the BGISEQ-500 PE50 data (0.0006%). For indels, BGISEQ-500 PE100 data resulted in worse performance with lower sensitivity (88.52%) than the HiSeq2500 PE150 data, with a sensitivity of 96.28%. In contrast, HiSeq2500 PE150 data shows a lower FPR (0.00032%) than BGISEQ-500 PE100 data (0.00069%). The BGISEQ-500 PE50 data resulted in a sensitivity of 70.93% and FPR of 0.00067%. The difference performances of indel calling might also be caused by read length difference (50 or 100 bp compared to 150 bp), in addition to sequencing quality, mapping accuracy, etc.

**Table 4:** Performances of variation calling of dataset*

Variant Type	Metrics	BGISEQ-500 PE50	BGISEQ-500 PE100	HiSeq2500 PE150
SNPs	True positive	3 006 132	3 071 579	3 084 449
	False positive	15 203	6907	4318
	False negative	186 825	121 379	108 508
	Precision	99.50%	99.78%	99.86%
	Sensitivity	94.15%	96.20%	96.60%
	FPR	0.00060%	0.00020%	0.00017%
	FNR	5.85%	3.80%	3.40%
indels	True positive	261 867	326 810	355 728
	False positive	16 931	22 246	7981
	False negative	107 311	42 391	13 751
	Precision	93.93%	93.63%	97.81%
	Sensitivity	70.93%	88.52%	96.28%
	FPR	0.00067%	0.00069%	0.00032%
	FNR	29.7%	11.48%	3.72%

*Above, the first four metrics are calculated using rtg-tools software. True positive (TP) is the number of SNPs that are found in the high-confidence reference dataset, false positive (FP) is the number of SNPs that are not found in reference dataset, and false negative (FN) is the number of SNPs that are found in high-confidence reference dataset but are not found in reference dataset. Precision is TP/(TP+FP)*100. Sensitivity is TP/(TP+FN)*100. FPR is FP/(all high-confident region length-TP-FN)*100, where high-confident region length equals 252 9164 928 bp, which comes from GIAB released high-confidence variants datasets [19]. FNR is FN/(FN+TP)*100.

Furthermore, to depict variation calling accuracy in different genomic regions, we compared the false negative rate (FNR), FPR, and sensitivity in different genome contexts given by GIAB (Fig. S1). For the coding sequences, data from the two platforms have similar FNR, FPR, and sensitivity (3.85% vs 2.52%, 0.00012% vs 0.00015%, and 96.15% vs 97.48%, respectively). For the regions that are difficult to sequence—including some of the promoters [15], substantially high GC content (>55%) regions, substantially low GC content (<30%) regions, regions with multiple variations (more than one variation within 50 bp), regions with compound variations, repeats, and segmental duplications—BGISEQ-500 data has a higher FNR, lower sensitivity, and lower FPR (Fig. S1).

## Discussion

Using the new sequencer, BGISEQ-500, we obtained one run of PE50 data and the other run of PE100 data. The raw data were ∼135.5 Gbp and ∼153.6 Gbp, respectively, and were generated from two chips (∼72 hours). Thus the sequencing throughput and turnaround time were comparable to HiSeq2500 sequencer Rapid mode v1 (∼80 Gbp per single flow cell and ∼40 hours). Both the single base quality and read quality (reflected by duplication rate, mapping rate, and unique mapping rate) were basically comparable to those of the HiSeq2500 data. Furthermore, the variation calling result was similar to that identified using similar amounts of HiSeq2500 data, further reflecting that the sequencer can be used in different research and applications. With future improvements in data quality, sequencing length, different and optimized insert sizes of the paired reads, and specially modified or designed software/bioinformatics tools, the performance could be further improved. In the meantime, the quality of the whole-genome sequencing data also reflected the feasibility of applying this sequencing platform to other sequencing purposes, including transcriptome, epigenome, metagenome, etc. From this first reference dataset of sequencing data from the BGISEQ-500 sequencer, we provided an overview and some basic information for the new sequencing platform. This dataset can serve as a reference for all the research using the BGISEQ-500 sequencing platform. And we anticipate that it help stimulate the further technical improvement and development of novel tools for accurately analyzing these data.

## Additional files

Additional file 1: Figure S1: Single nucleotide polymorphism stratification performance between BGISEQ-500 and HiSeq2500. We used stratification files from the Global Alliance for Genomics and Health (GA4GH) Benchmarking Team and the Genome in a Bottle Consortium that were intended as a standard resource of bed files for use in stratifying true positive, false positive, and false negative variant calls into different categories. We list detailed information about different regions.

Additional file 1: Table S1: Performances of variation calling under different filtering threshold conditions.

## List of abbreviations

bp - base-pair

dATP - deoxyadenosine triphosphate

dTTP - deoxythymidine triphosphate

DNBs - DNA nanoballs

FNR - false negative rate

FPR - false positive rate

GIAB - Genome in A Bottle

PCR - polymerase chain reaction

PE50 - pair-end 50 bp

PE100 - pair-end 100 bp

SNPs - Single Nucleotide Polymorphisms

indels - insertions and deletions

## Availability of supporting data

The BGISEQ-500 sequences described in this article are available in the GigaDB repository (PE 50 [16] and PE 100 [17]) and the European Nucleotide Archive under accession number ERP017158. This GigaDB entry also contains examples of the raw image data, including images of all the sequencing cycles in a small region and images of the first and last 10 cycles of the whole flow cell [16]. Future data will also be updated via the GigaDB repository with versions indicated.

## Conflicts of interest

J.H., Y.X., S.Q., X.M., H.C., T.Y., N.S., Z.Y., and S.G. are involved in the beta test of the BGISEQ-500 sequencer. X. Liang, C.G., Y.L., H.L., H.J., X. Liu, and F.M. are involved in the BGISEQ-500 sequencer development, library construction technology optimization, base calling software development, or alpha and beta tests.

## Authors’ contributions

J.H., Z.Y., F.M., and S.G. designed the project. Y.X., S.Q., and C.G. conducted sample preparation and sequencing library construction. H.L., X.M., H.C., T.Y., and N.S. conducted sequencing. X. Liang, J.R., J.W., Y.L., X. Liu, H.J., J.R., J.W., W.Z., Y.C., and S.L. conducted data analysis. X. Liu, X. Liang, Y.L., C.G., H.L., J.H., and H.J. wrote the manuscript.

## Supplementary Material

GIGA-D-16-00103_Original_Submission.pdf

GIGA-D-16-00103_Revision_1.pdf

GIGA-D-16-00103_Revision_2.pdf

Response_to_reviewer_comments_Original_Submission.pdf

Response_to_reviewer_comments_Revision_1.pdf

Reviewer_1_Report_(Original_Submission).pdf

Reviewer_2_Report_(Original_Submission).pdf

Reviewer_2_Report_(Revision_1).pdf

Supplemental material Additional file 1: Figure S1: Single nucleotide polymorphism stratification performance between BGISEQ-500 and HiSeq2500. We used stratification files from the Global Alliance for Genomics and Health (GA4GH) Benchmarking Team and the Genome in a Bottle Consortium that were intended as a standard resource of bed files for use in stratifying true positive, false positive, and false negative variant calls into different categories. We list detailed information about different regions.
Additional file 1: Table S1: Performances of variation calling under different filtering threshold conditions.
