# Erratum to: A reference human genome dataset of the BGISEQ-500 sequencer

**DOI:** 10.1093/gigascience/giy144

**Published:** 2018-11-30

**Authors:** Jie Huang, Xinming Liang, Yuankai Xuan, Chunyu Geng, Yuxiang Li, Haorong Lu, Shoufang Qu, Xianglin Mei, Hongbo Chen, Ting Yu, Nan Sun, Junhua Rao, Jiahao Wang, Wenwei Zhang, Ying Chen, Sha Liao, Hui Jiang, Xin Liu, Zhaopeng Yang, Feng Mu, Shangxian Gao

**Affiliations:** 1 National Institutes for food and drug Control (NIFDC), Beijing 100050, P. R. China; 2 BGI-Shenzhen, Shenzhen 518083, P. R. China; 3 State Food and Drug Administration Hubei Center for Medical Equipment Quality Supervision and Testing, Wuhan 430000, P. R. China; 4 BGI-Qingdao, Qingdao 266555, China

We found several inaccurate descriptions in our recently published paper, “A reference human genome dataset of the BGISEQ-500 sequencer” [1], thus we would like to make the following corrections.

In the Background section, the full name of cPAS should be “combinatorial probe-anchor synthesis” [2] rather than “combined primer anchor synthesis”. So the following sentence should be modified:

1. Sentence “… sequencing library construction and combined primer anchor synthesis (cPAS) for sequencing.” should be changed to “… sequencing library construction and combinatorial probe-anchor synthesis (cPAS) for sequencing.”.
  In the Background-Sequencing section, the mentioned sequencing technology should be “rolling circle amplification (RCA)” [2] instead of “PCR amplification”. The RCA step is one of Complete Genomics^TM^ core technologies, which is different from PCR amplification. The key advantage of the DNB technology is that every replicated copy is produced from the original genomic fragment, instead of PCR-utilizing sequencing technologies, in which one amplifies from the already amplified template. Thus two sentences should be modified accordingly:
2. Sentence “… single-strand circular DNA library was first PCR-amplified for 10 minutes….” should be changed to “… single-strand circular DNA library was first subjected to rolling circle amplification (RCA) for 10 minutes…”.
3. Sentence “After the PCR reaction, 20 μl DNBs stopping buffer was added to terminate the PCR reaction” should be changed to “After the RCA reaction, 20 μl DNBs stopping buffer was added to terminate the RCA reaction”.

For clarity we have uploaded detailed and corrected protocols of the sequencing library preparation [3] and sequencing [4] steps to protocols.io.
