# Supplementary material for: A reference human genome dataset of the BGISEQ-500 sequencer
Source: Gigascience. 2017 Apr 1;6(5):1–9. doi: 10.1093/gigascience/gix024 (PMC5467036; doi:10.1093/gigascience/gix024)
Supplement: GIGA-D-16-00103_Original_Submission.pdf [file gix024_GIGA-D-16-00103_Original_Submission.pdf]

Manuscript

Click here to view linked References

Click here to download Manuscript WGSDataNoteV1.0_SCE.docx

A reference human genome dataset of the BGISEQ-500 sequencer

Jie Huang[1*], Xinming Liang[2*], Yuankai Xuan[3*], Chunyu Geng[2], Yuxiang Li[2], Haorong Lu[2], Shoufang Qu[1], Xianglin Mei[3], Hongbo Chen[1], Ting Yu[1], Nan Sun[1], Hui Jiang[2], Xin Liu[2], Zhaopeng Yang[1#], Feng Mu[2#] and Shangxian Gao[1#]

*These authors contribute equally to the article

#Correspondence: Shangxian Gao, gaoshangxian@126.com, Feng Mu mufeng@genomics.cn and Zhaopeng Yang yangzp@nifdc.org.cn

[1] National Institutes for food and drug Control (NIFDC), Beijing 100050, P. R. China

[2] BGI-Shenzhen, Shenzhen 518083, P. R. China

[3] State Food and Drug Administration Hubei Center for Medical Equipment Quality Supervision and Testing, Wuhan 430000, P. R. China

Abstract

Background: BGISEQ-500 sequencer is a new desktop sequencer developed by BGI. Using DNA nanoballs (DNB) and combinational probe-anchor synthesis (cPAS) developed from Complete Genomics^{TM} sequencing technology, it generates short reads at a large scale, which can help fulfill the growing demands for sequencing.

Findings: Here, we present the first human whole genome sequencing dataset from the BGISEQ-500. The dataset was generated by sequencing the widely used Genome in a Bottle Consortium cell line, HG001 (NA12878) in one sequencing run. And the sequencing data were ~1,000 million paired sequences with the length of 50 bp (PE50). We also include examples of the raw images from the sequencer for reference. Finally, we carried out variation calling based on the dataset and compared it that identified from similar amount of publicly available HiSeq2500 data and the previously identified high confident variations.

Conclusions: We found that despite the shorter length of the BGISEQ-500 data, the data quality was comparable to data from other sequencing platforms. For SNP calling, BGISEQ-500 dataset had relatively lower false positive rate and sensitivity. We also found some discrepancies of the BGISEQ-500 data, especially for indel calling, which would require further improving of the data quality as well as the data analysis tools. Our dataset can serve as the reference dataset providing basic information not just for future developing, but also for all the researches and applications based on the new sequencing platform.

Keywords: Genomics, sequencing, second generation sequencing, BGISEQ-500

Data Description

Massively parallel sequencing technologies (also called as the second generation sequencing) generate large amount of data with lower cost, shorter reads and higher single base error rate compared to Sanger sequencing technology [1]. With the large amount of data and well-developed analysis tools, second generation sequencing data can be used to effectively and accurately identify genomic variations in a much more cost effective manner than previous sequencing technologies [2] thus it has been widely applied in both researches and applications [3]. Currently there are several commercially available second generation sequencing platforms with different performances and data features [4, 5]. With more and more research and applications to apply sequencing to, new sequencing platforms are being developed to continue to move the field forward. BGISEQ-500 is a new low cost desktop sequencer first released by BGI in October, 2015. It was developed based on Complete Genomics™ sequencing technologies, and applies DNA nanoball (DNB) technology [6] for sequencing library construction and combined primer anchor synthesis (cPAS) for sequencing. We present here a reference dataset generated from the BGISEQ-500 sequencer, including example of the raw images and the final sequences. We also conducted variation calling using this dataset and compared the variation calling result to that from other sequencers. This dataset can be served as a useful reference for community to develop bioinformatics methods and sequencing based applications on this new sequencing platform.

DNA preparation

NA12878 cell line (RRID: CVCL_7526) genomic DNA was ordered from Coriell Institute, and consisted of 50ug per tube. The genomic DNA was quantified by Qubit 3.0 fluorometer (Life Technologies, USA) and the integrity was qualified on a 2% agarose gel to make sure the genomic DNA was larger than 23 kb and not substantially degraded.

Sequencing library preparation

For the sequencing library construction, the NA12878 genomic DNA was fragmented by a Covaris LE220 sonicator (Covaris, USA) to 50 bp~800 bp DNA fragments according to the manufacturer's instructions. The fragmented DNA was further selected to 100 bp~300 bp fragments by AMPure XP beads (Beckman Coulter, USA). The selected DNA fragments were then repaired to obtain a blunt end and modified at the 3' end to get a dATP sticky-end. The dTTP tailed adapter sequence was ligated to both ends of the DNA fragments. The ligation product was then amplified for 8 cycles and subjected to the single strand circularization process. The PCR product was heat-denatured together with a special molecule which was reverse complemented to one special strand of the PCR product, and the single strand PCR product was ligated using DNA ligase. The remaining linear molecule was digested with the exonuclease, thus finally we obtained the single strand circle DNA library (Figure 1a).

Sequencing

We conducted sequencing according to the BGISEQ-500 protocol (Figure 1b). There were three steps including making DNBs, loading DNBs and sequencing. For making DNBs, 6 ng single strand circle DNA library was first subjected to rolling circle amplification (RCA) for 10 minutes in an 80 ul reaction volume with pure water, buffer and DNB polymerase. After the RCA reaction, 20 ul DNBs stopping buffer was added to stop the RCA reaction. Finally, we used the Qubit® ssDNA Assay Kit to quantify the DNBs on Qubit® Fluorometer (concentration $\geq$ 10 ng/μL).

For loading DNBs, we first added 33ul DNBs loading buffer to the DNBs product, and the mixture was placed on the BGIDL-50 (the sample preparation machine). Then we selected the DNBs loading process (Version: sample load 2.0) to load DNB onto the sequencing chip, which included 96 minutes' loading time and 30 minutes' incubation at room temperature.

Finally, we applied the BGISEQ-500 protocol to conduct sequencing. We selected sequence control software Version 1.1.0.10003, sequence process Version 1.0.06 and Zebracall Version 0.5.0.13875 (the base calling software, for which a detailed

description can be found in the next section) for sequencing. Sequencing was initiated after the sequencing reagents pre-loaded and sequencing chip installed, and this process was finished in ~72 hours.

Base calling and raw images

During sequencing, four channels of 16-bit grey scale images were captured by high resolution sCMOS with ~5.5 million pixels per image. About ~570K DNBs were loaded onto the grid-patterned arrays of spots which were photolithographically etched and surface modified on the sequencing chip. The spots were illuminated by the lasers with different wavelengths. Spots from the neighboring channel would also be observed due to crosstalk effect.

The sequences of DNBs were base-called by the software Zebracall (base calling software developed for BGISEQ-500). After background subtraction and registration of images from 4 channels, intensities of DNBs were extracted according to a template of grid-pattern. Correction within channels and neighbor cycles was applied to increase the quality and stabilization. After all correction step, reads were converted into FASTQ format with Phred+33 quality score. An example dataset of the images was included and the base calling process is illustrated in Figure 2.

Results

Sequencing data summary

The sequencing data consists of two lanes, and the total raw reads were ~2.7×10^9 pairs (Table 1), and for each paired reads, the sequencing length of each read was 50 bp. We then conduct data filtering to filter low quality reads which had more than 10% bases with sequencing quality lower than 10, and reads which had more than 1% Ns (ambiguous bases). In this way, we filtered ~12.3% low quality raw reads thus obtained ~2.4×10^9 pairs of clean reads which was ~118.9 G bp. We then used FastQC [7] to conduct quality control for the clean reads (Figure 3). We also used a subset (from 8 sequencing libraries and 16 lanes, 2×148 reads, ~98.5 G bp data) of Illumina HiSeq2500 reads of the same cell line from GIAB (Genome in a Bottle) [8]. After the same data filtering process which filtered ~7.95% raw reads, we carried out the same quality control for comparison (Figure 3).

Variation calling and False positive/negative ratios estimation

In order to further depict the data quality and test applications of the new sequencing platform, we carried out variation calling using this dataset. We adapted the currently widely used pipeline (mapping using BWA [9] and variation calling using GATK [10-12], an illustration of the pipeline and key parameters can be found in Figure 4a) for variation calling. We observed higher mapping rate, similar sequencing coverage (in the condition of similar sequencing depth) and similar sequencing uniformity in the dataset comparing to the HiSeq data (Table 2). And the lower unique mapping rate probably reflected the shorter read length of the dataset (2×50 bp comparing to 2×150 bp). We also observed relatively higher duplication rate and mismatch rate in the dataset comparing to the HiSeq data (Table 2).

In total, we identified 3,451,124 SNPs and 554,568 indels (insertions and deletions) using the dataset, which were less than those identified using the HiSeq data

(3,621,362 SNPs and 686,697 indels) (Table 3). The SNPs were similar to those identified from HiSeq data in different features including dbSNP rate, proportion of SNPs in different regions related to genes and Ti/Tv (transition-transversion) ratio, which indirectly reflected the SNP accuracy. We also observed similar situation for indels.

Further to assess the accuracy of the variations, we used the high confident region (~2.5 Gb human genome region with explicit genotypes, and 3,154,902 of them were SNPs) previously identified in NA12878 [13]. In the high confident region, 2,975,482 SNPs were also identified using the BGISEQ-500 dataset (Figure 4b), resulting in a rough estimation of the sensitivity to be 94.3%, comparing to 97.2% for the HiSeq dataset. For the 179,420 high confident SNPs in region which were not identified using the BGISEQ-500 data, we found significantly lower sequencing depth comparing to the identified high confident SNPs (Wilcox rank sum test, $P<0.01$) (Figure 4c), indicating that sequencing depth should be one of the reasons for lower sensitivity and improving sequencing depth would probably improve the sensitivity. In the high confidence region, we identified 11,934 SNPs which were not included in the high confidence SNPs thus should probably be false positives. In this way we estimated the false positive rate to be 0.4%, comparing to 0.07% of the HiSeq data (2,213 SNPs in the high confident region but not in the high confident SNPs). However, for indels, we observed similar false positive rate (0.38% for BGISEQ-500 and 0.14% for HiSeq data) but much lower sensitivity (69.2% comparing to 93.0% of the HiSeq data), indicating a discrepancy in the dataset in indel identification.

Discussion

Using the new sequencer, BGISEQ-500, we obtained paired reads with sequencing length of 50 bp at both ends. The raw data was ~135.5 G bp and was generated in two sequencing lanes of a single sequencing run in ~72 hours. Thus the sequencing throughput and turnaround time were comparable to HiSeq2500 sequencer (~80 G bp per lane and ~40 hours). The single base quality, reads quality reflected by duplication rate, mapping rate and unique mapping rate, were therefore comparable to those of the data from other sequencing platforms. Furthermore, the SNP calling result was similar to that identified using similar amounts of HiSeq data, further reflected that the sequencer can be used in different researches and applications. In the meantime, we also observe some discrepancies in the dataset. Especially for the current sequencing length, the indel identification and probably structural variation calling might be problematic. Future improvements over data quality, sequencing length, different and optimized insert sizes of the paired reads, as well as specially modified or designed software/bioinformatics tools are necessary. From this first reference dataset of sequencing data from BGISEQ-500 sequencer, we provided an overview and some basic information for the new sequencing platform. This dataset can serve as reference for all the researches using the BGISEQ-500 sequencing platform. And we anticipate it to help stimulating the further technical improving, and developing of novel tools for accurately analyzing the data.

Availability of supporting data

The BGISEQ-500 dataset (sequences) described in this article is available in the GigaDB repository (ftp://user14@climb.genomics.cn/BGISEQ-500_WGS/), and the European Nucleotide Archive under accession number PRJEB15427. This GigaDB repository for this article also contains examples of the raw image data including images of all the sequencing cycles in a small region and images of the first and last 10 cycles of the whole flowcell. Future data which are to be generated will also be updated in this GigaDB repository with versions indicated.

Abbreviations

DNBs: DNA nanoballs; SNPs: Single Nucleotide Polymorphisms; indels: insertions and deletions

Competing interests

JH, YX, SQ, XM, HC, TY, NS, ZY and SG are involved in the beta test of the BGISEQ-500 sequencer. X Liang, CG, YL, HL, HJ, X Liu and FM are involved in the BGISEQ-500 sequencer developing, library construction technology optimization, base calling software developing, or alpha and beta tests.

Authors' contributions

JH, ZY, FM and SG designed the project. YX, SQ and CG conducted sample preparation and sequencing library construction. HL, XM, HC, TY and NS conducted sequencing. X Liang, YL, X Liu and HJ conducted data analysis. X Liu, X Liang, YL, CG, HL, JH and HJ wrote the manuscript.

Acknowledgements

This work was supported by following funding: the National High Technology Research and Development Program ("863" Program) of China (Project No. 2011AA02A115), the Technology Innovation and Developing Plan of Shenzhen (Project No. CXZZ20140904154910774).

Author details

[1] National Institutes for food and drug Control (NIFDC), Beijing 100050, P. R. China

[2] BGI-Shenzhen, Shenzhen 518083, P. R. China

[3] State Food and Drug Administration Hubei Center for Medical Equipment Quality Supervision and Testing, Wuhan 430000, P. R. China

References

1. Metzker ML: **Sequencing technologies - the next generation**. *Nat Rev Genet* 2010, **11**(1):31-46.
2. Wang J, Wang W, Li R, Li Y, Tian G, Goodman L, Fan W, Zhang J, Li J, Guo Y *et al*: **The diploid genome sequence of an Asian individual**. *Nature* 2008, **456**(7218):60-65.
3. Goodwin S, McPherson JD, McCombie WR: **Coming of age: ten years of**

next-generation sequencing technologies. *Nat Rev Genet* 2016, **17**(6):333-351.

4. Mardis ER: **Next-generation sequencing platforms**. *Annu Rev Anal Chem (Palo Alto Calif)* 2013, **6**:287-303.
5. Quail MA, Smith M, Coupland P, Otto TD, Harris SR, Connor TR, Bertoni A, Swerdlow HP, Gu Y: **A tale of three next generation sequencing platforms: comparison of Ion Torrent, Pacific Biosciences and Illumina MiSeq sequencers**. *BMC Genomics* 2012, **13**:341.
6. Drmanac R, Sparks AB, Callow MJ, Halpern AL, Burns NL, Kermani BG, Carnevali P, Nazarenko I, Nilsen GB, Yeung G *et al*: **Human genome sequencing using unchained base reads on self-assembling DNA nanoarrays**. *Science* 2010, **327**(5961):78-81.
7. Andrews S: **FastQC, a quality control tool for high throughput sequence data**. 2016.
8. Zook JM, Catoe D, McDaniel J, Vang L, Spies N, Sidow A, Weng Z, Liu Y, Mason CE, Alexander N *et al*: **Extensive sequencing of seven human genomes to characterize benchmark reference materials**. *Sci Data* 2016, **3**:160025.
9. Li H, Durbin R: **Fast and accurate short read alignment with Burrows-Wheeler transform**. *Bioinformatics* 2009, **25**(14):1754-1760.
10. McKenna A, Hanna M, Banks E, Sivachenko A, Cibulskis K, Kernytsky A, Garimella K, Altshuler D, Gabriel S, Daly M *et al*: **The Genome Analysis Toolkit: a MapReduce framework for analyzing next-generation DNA sequencing data**. *Genome Res* 2010, **20**(9):1297-1303.
11. DePristo MA, Banks E, Poplin R, Garimella KV, Maguire JR, Hartl C, Philippakis AA, del Angel G, Rivas MA, Hanna M *et al*: **A framework for variation discovery and genotyping using next-generation DNA sequencing data**. *Nat Genet* 2011, **43**(5):491-498.
12. Van der Auwera GA, Carneiro MO, Hartl C, Poplin R, Del Angel G, Levy-Moonshine A, Jordan T, Shakir K, Roazen D, Thibault J *et al*: **From FastQ data to high confidence variant calls: the Genome Analysis Toolkit best practices pipeline**. *Curr Protoc Bioinformatics* 2013, **43**:11 10 11-33.
13. Zook JM, Chapman B, Wang J, Mittelman D, Hofmann O, Hide W, Salit M: **Integrating human sequence data sets provides a resource of benchmark SNP and indel genotype calls**. *Nat Biotechnol* 2014, **32**(3):246-251.

Figure Legends

Figure 1. Flowchart of library construction and sequencing. The library construction includes fragmentation, size selection, end-repair and A-tailing, adaptor ligation and PCR amplification and splint circularization (**a**). The sequencing includes making DNBs, loading DNBs and sequencing (**b**).

Figure 2. Raw image data processing on the BGISEQ-500 platform. a. Registration of images from different channels. Relative coordinates will be calculated according to the pattern layout of DNBs. **b. Intensity correction between channels and cycles.** In order to correct the interference by optical and chemical, correction was applied to different channels and the neighbor cycles. **c. Connecting called bases to FASTQ.** Bases from all cycles will be collected and converted to FASTQ format. Phred score calculation and statistics will be applied during the conversion.

Figure 3. Quality control of the dataset after data filtering. a) Base-wise quality scores of the BGISEQ-500 reads. For each position along the reads, the quality scores of all reads were used to calculate the mean, median and quantile values thus the box plot can be shown. **b) Base-wise quality scores of the HisSeq2500 reads.** The reads were 150 bp long and pair-end reads. **c) The quality score distribution of BGISEQ-500 and HiSeq2500 data.** The quality scores of BGISEQ-500 reads were lower than the HiSeq2500 reads. **d) GC content distribution of the BGISEQ-500 and HiSeq2500 data.** The GC content distributions were similar.

Figure 4. Variation calling based on the dataset and comparison of the SNPs. a) Flowchart of the variation calling process. The major steps included data filtering, alignment and variation calling, and the major parameters are also indicated. **b)** and **c) Estimation of the SNP/indel detection sensitivity and false positive rate.** The high

confident regions were previously determined 2.5 Gb region with explicit genotypes (indicated as the grey box), and within these regions there were variations of which the genotypes were different from the reference (indicated as the light red box). The SNPs/indels identified using BGISEQ-500 data were indicated as the green box while those of HiSeq2500 data were indicated as the red box.

Tables

Table 1. Summary of the dataset*.

	Read (×10^9)	Bases (Gbp)	Percentage of Ns	GC content	>Q20	>Q30	Error rate
**L1R1**	0.70	35.03	0.15%	41.49%	98.14%	91.48%	0.30%
**L1R2**	0.70	35.03	0.18%	41.55%	94.08%	83.21%	0.69%
**L1**	1.40	70.06	0.16%	41.59%	96.11%	87.34%	0.50%
**L2R1**	0.66	32.77	0.09%	41.57%	97.89%	90.71%	0.27%
**L2R2**	0.66	32.77	0.16%	41.61%	93.87%	82.69%	0.68%
**L2**	1.31	65.55	0.12%	41.64%	95.88%	86.70%	0.48%

*This dataset was from a single run of the BGISEQ-500 sequencer. It has two lanes (Lane 1 denoted as L1 and Lane 2 denoted as L2, respectively), and the reads were paired (Read 1 denoted as R1 and Read 2 denoted as R2, respectively) with 50 bp in length each (2×50 bp). For the read number and bases, some statistics of the two reads probably doesn't add up to the statistics of the lane just because of rounding. '>Q20/Q30 percentage' indicates the percent of bases with quality score (-10×lg(error rate)) higher than 20 and 30 (indicating error rates of 1% and 1‰ respectively). In the final column, error rate was the average error rate of all the bases.

Table 2. Mapping statistics of the dataset*.

**Metrics**	**BGISEQ-500**	**HiSeq2500**
Clean reads	2,378,725,921	665,279,408
Clean bases(bp)	118,936,296,050	98,461,352,384
Mapping rate	97.87%	95.74%
Unique rate	93.17%	97.22%
Duplicate rate	6.26%	0.98%
Mismatch rate	0.34%	0.27%
Average sequencing depth	37.57	31.94
Coverage	99.28%	99.01%
Coverage at least 4×	98.90%	98.46%
Coverage at least 10×	97.97%	97.34%

Coverage at least 20×	95.78%	91.61%

*The BGISEQ-500 data were 50 bp paired reads while the HiSeq2500 data were 150 bp paired reads. The statistics shown here are calculated based on the clean reads (raw reads after filtering, the two platforms' data went through the same filtering process). Unique mapping rate indicates proportion of reads with unique alignment in the genome.

Table 3. Variation statistics of the dataset*.

Sample	BGISEQ-500	HiSeq2500
Total	3,451,124	3,621,362
1000genome and dbsnp141	3,242,083	3,342,070
1000genome specific	1,260	1,712
dbSNP141 specific	180,935	246,684
dbSNP rate	99.19%	99.10%
Novel SNPs	26,846	30,896
Homozygous SNPs	1,426,328	1,475,262
Heterozygous SNPs	2,024,796	2,146,100
Synonymous SNPs	19,880	20,786
Ti/Tv	2.05	2.04
dbSNP Ti/Tv	2.06	2.05
Novel Ti/Tv	0.89	1.08

*1000genome and dbsnp141 equals the number of SNPs that are found in both 1000 genome and dbSNP databases, 1000genome specific equals the number of SNPs that are only found in 1000 genomes database. dbSNP rate equals the number of SNPs found in dbSNP database/total detected SNPs. Novel SNP equals the number of SNPs that are not found in SNP database. Ti/Tv equals the ratio of SNP type are transition/SNP type are transversion. TP (True Positive) equals the number of SNPs that are found in high-confidence reference dataset, FP (False Positive) equals the number of SNPs that are not found in reference dataset, FP equals FP/(FP+TN), which TN equals the number of positions

that are same as reference. FN equals the number of SNPs that are not detected but are recorded in reference dataset, FN rate equals FN/(FN+TP). Sensitivity equals TP/(TP+FN). PPV is short for positive predictive value, which equals TP/(TP+FP).

 Click here to download Figure Figure 1.pdf

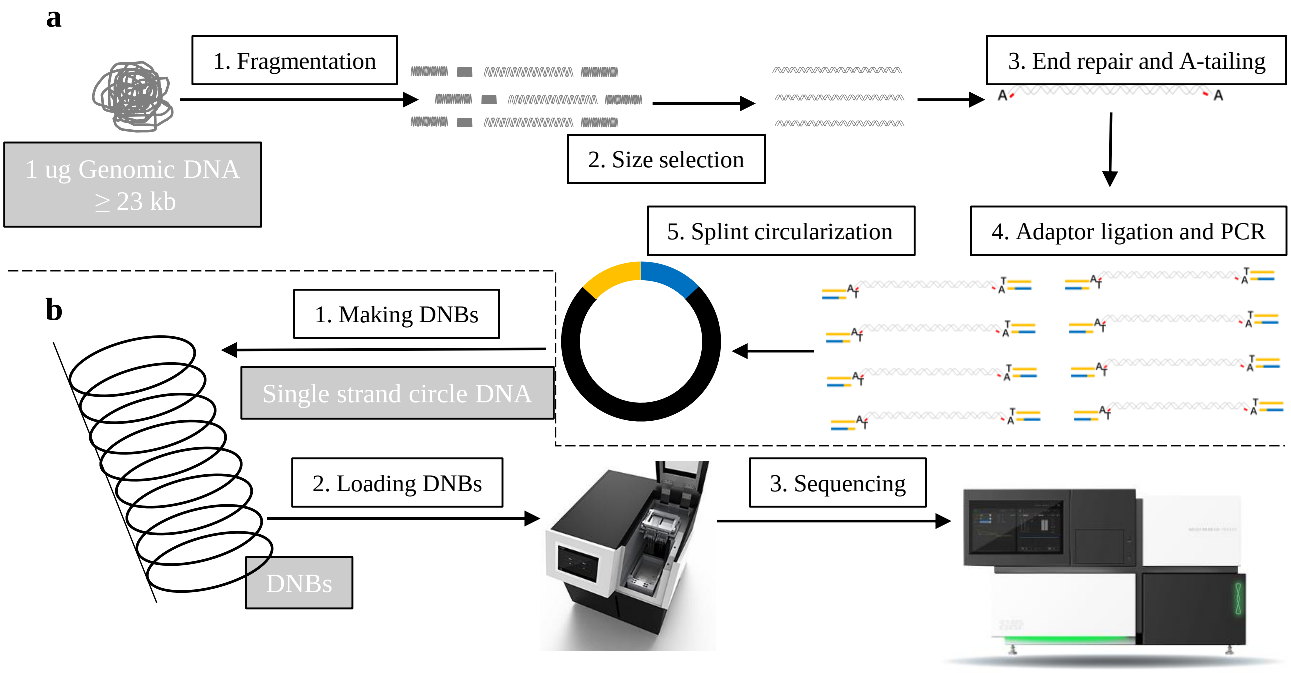

Figure 2

Click here to download Figure Figure 2.pdf

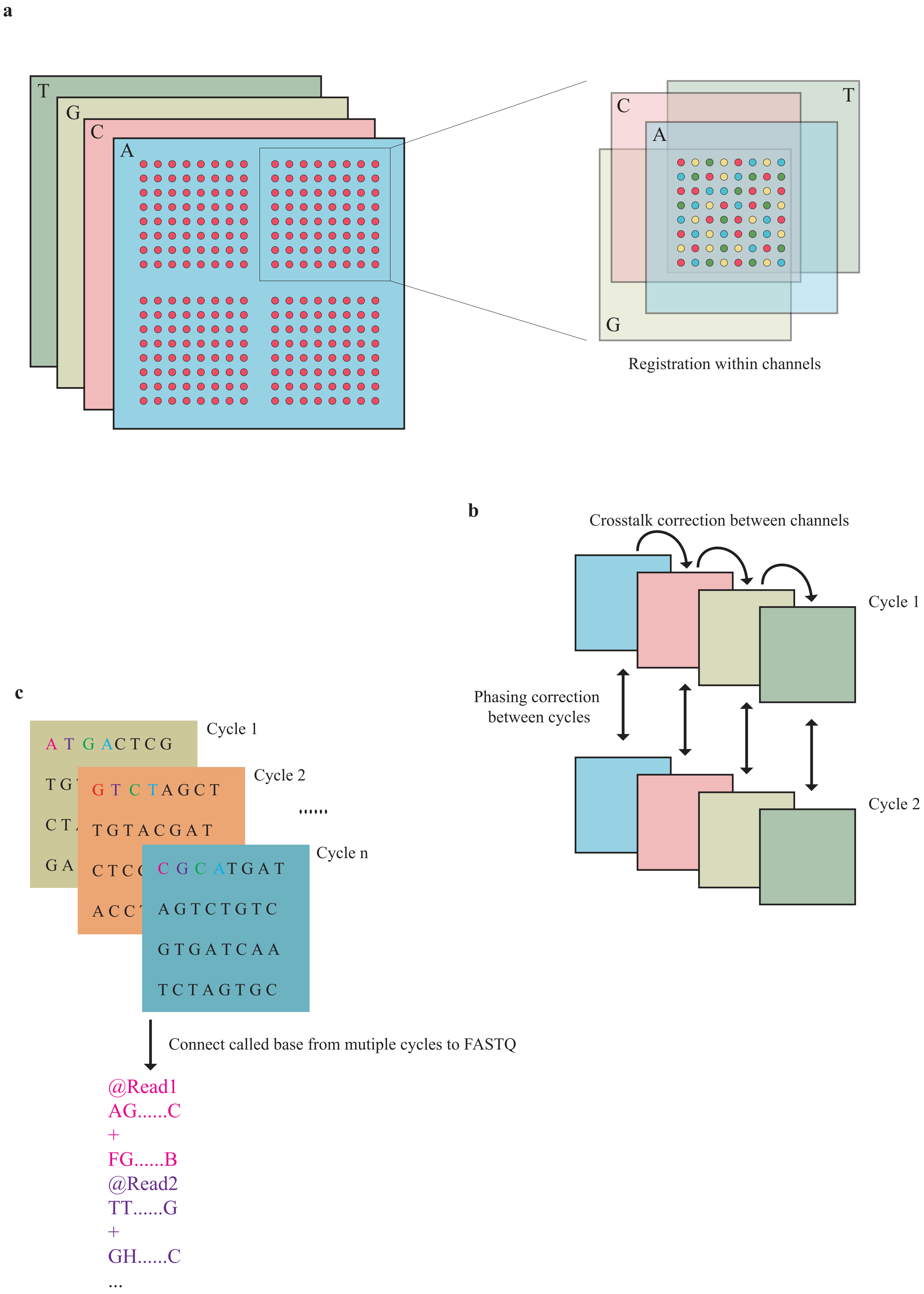

Figure 3

Click here to download Figure Figure 3.pdf

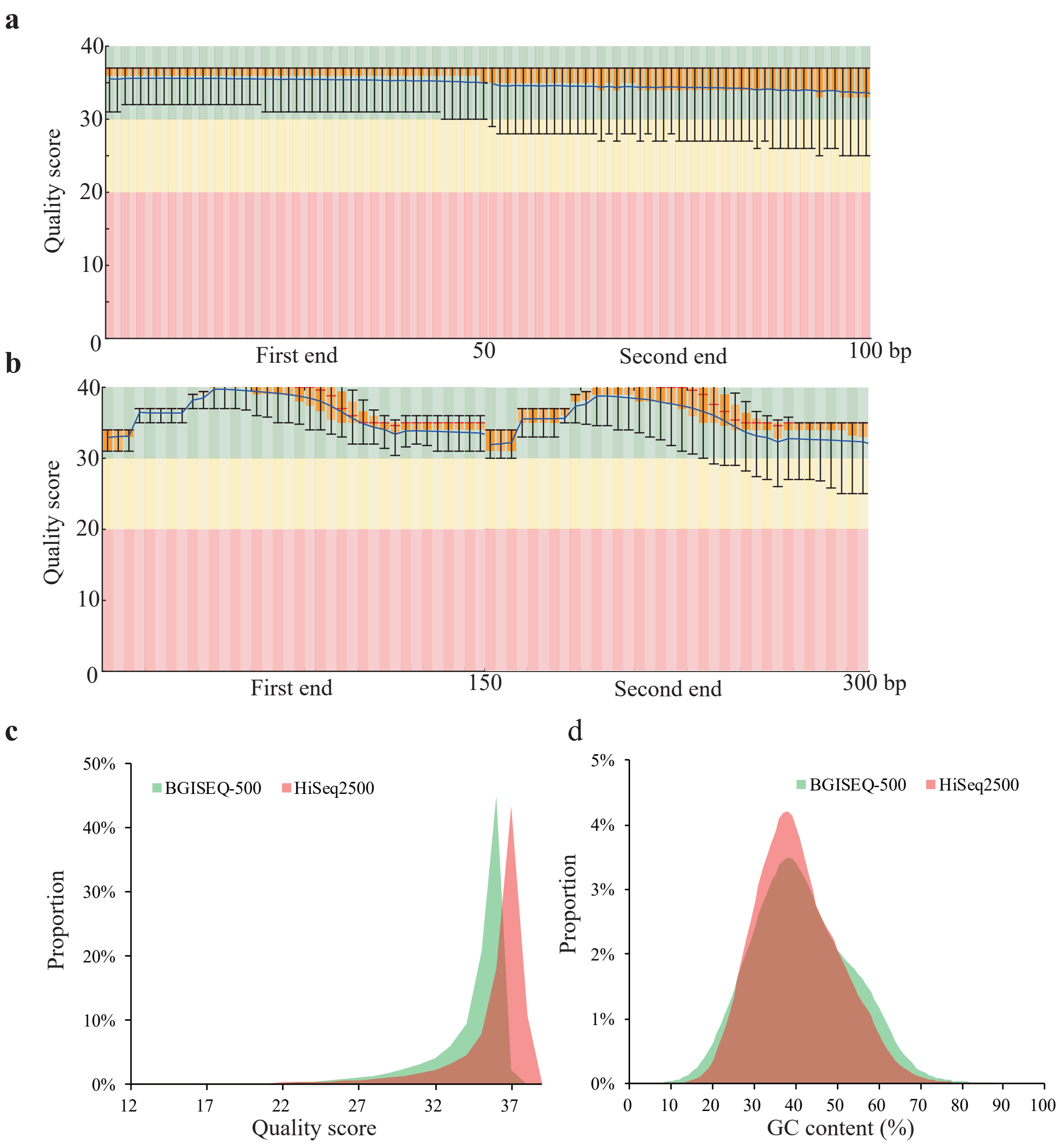

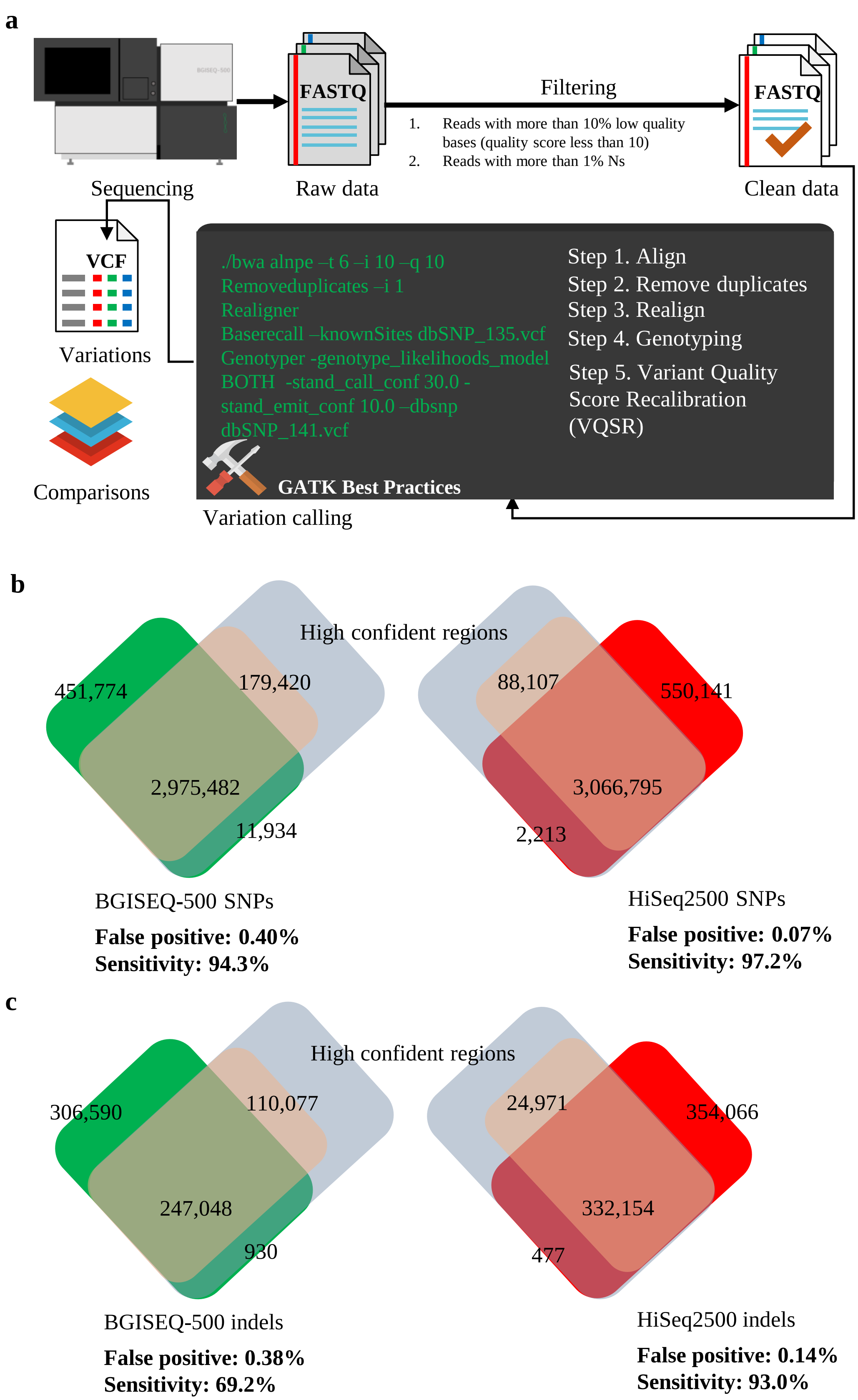
a
FASTQ
Filtering
1. Reads with more than 10% low quality bases (quality score less than 10)
2. Reads with more than 1% Ns
FASTQ
Sequencing
Raw data
Clean data
VCF
Variations
Comparisons
./bwa alnpe –t 6 –i 10 –q 10
Removeduplicates –i 1
Realigner
Baserecall –knownSites dbSNP_135.vcf
Genotyper -genotype_likelihoods_model BOTH -stand_call_conf 30.0 -stand_emit_conf 10.0 –dbsnp dbSNP_141.vcf
Step 1. Align
Step 2. Remove duplicates
Step 3. Realign
Step 4. Genotyping
Step 5. Variant Quality Score Recalibration (VQSR)
GATK Best Practices
Variation calling
b
High confident regions
451,774
179,420
2,975,482
11,934
88,107
550,141
3,066,795
2,213
BGISEQ-500 SNPs
False positive: 0.40%
Sensitivity: 94.3%
HiSeq2500 SNPs
False positive: 0.07%
Sensitivity: 97.2%
c
High confident regions
306,590
110,077
247,048
930
24,971
354,066
332,154
477
BGISEQ-500 indels
False positive: 0.38%
Sensitivity: 69.2%
HiSeq2500 indels
False positive: 0.14%
Sensitivity: 93.0%

 Click here to download Personal Cover CoverLetter.docx

Dear Editor,

We are now submitting a manuscript entitled ***A reference human genome dataset of the BGISEQ-500 sequencer*** for you to consider as a data note in *GigaScience*. In this manuscript, we have for the first time presented the sequencing data from the new sequencing platform BGISEQ-500. We obtained ~30× data of the commercially available cell line NA12878 from the new sequencing platform, and the sequencing data were 50 bp in length and paired (50 PE). We compared the data quality to similar amount of HiSeq sequencing data of the same cell line previously generated by the Genome in A Bottle consortium (GIAB), which were of high quality. Furthermore, we used the BGISEQ-500 dataset to conduct the variation calling, and compared the variation calling results identified from the HiSeq dataset. We found good sequencing quality of the BGISEQ-500 data, and good sensitivity and accuracy for variation calling, although some discrepancies were also observed indicating directions for future improvements.

We think that the new platform can be widely applied in both researches and applications, thus the comprehensive assessment of the data quality would be very valuable and will serve as reference for many other researches using the new platform, providing important information to a broad audience. In the meantime, further improvement of the data quality would also refer to the current analysis conducted in this manuscript thus we think it would fit the requirement of *GigaScience*. We hope that you will also find the manuscript potential interesting and consider for sending out for review. Any further suggestions are welcome and we can do further revision if necessary.

Our submission includes (1) the main manuscript file containing the main text, 3 tables, and 4 figures, along with the comprehensive dataset.

Yours sincerely,

Xin Liu, Ph. D.
Research Scientist, BGI-Shenzhen, Shenzhen 518083, PRC.
Email: liuxin@genomics.cn
