# Supplementary material for: A reference human genome dataset of the BGISEQ-500 sequencer
Source: Gigascience. 2017 Apr 1;6(5):1–9. doi: 10.1093/gigascience/gix024 (PMC5467036; doi:10.1093/gigascience/gix024)
Supplement: GIGA-D-16-00103_Revision_2.pdf [file gix024_GIGA-D-16-00103_Revision_2.pdf]

Manuscript Click here to download Manuscript Manuscript..docx

A reference human genome dataset of the BGISEQ-500 sequencer

Jie Huang[1*], Xinming Liang[2*], Yuankai Xuan[3*], Chunyu Geng[2], Yuxiang Li[2], Haorong Lu[2], Shoufang Qu[1], Xianglin Mei[3], Hongbo Chen[1], Ting Yu[1], Nan Sun[1], Junhua Rao[2], Jiahao Wang[4], Wenwei Zhang[2], Ying Chen[2], Sha Liao[2], Hui Jiang[2], Xin Liu[2], Zhaopeng Yang[1#], Feng Mu[2#] and Shangxian Gao[1#]

*These authors contribute equally to the article

#Correspondence: Shangxian Gao, gaoshangxian@126.com, Feng Mu mufeng@genomics.cn and Zhaopeng Yang yangzp@nifdc.org.cn

[1] National Institutes for food and drug Control (NIFDC), Beijing 100050, P. R. China

[2] BGI-Shenzhen, Shenzhen 518083, P. R. China

[3] State Food and Drug Administration Hubei Center for Medical Equipment Quality Supervision and Testing, Wuhan 430000, P. R. China

[4] BGI-Qingdao, Qingdao 266555, China

Abstract

Background: BGISEQ-500 is a new desktop sequencer developed by BGI. Using DNA nanoball (DNB) and combinational probe anchor synthesis (cPAS) developed from Complete Genomics^{TM} sequencing technologies, it generates short reads at a large scale.

Findings: Here, we present the first human whole genome sequencing dataset of BGISEQ-500. The dataset was generated by sequencing the widely used cell line, HG001 (NA12878) in two sequencing runs of paired-end 50 bp (PE50) and two sequencing runs of paired-end 100 bp (PE100). We also include examples of the raw images from the sequencer for reference. Finally, we identified variations using this dataset, estimated the accuracy of the variations and compared to that of the variations identified from similar amounts of publicly available HiSeq2500 data.

Conclusions: We found similar SNP detection accuracy for the BGISEQ-500 PE100 data (false positive rate, FPR 0.00020%, and sensitivity 96.20%) comparing to the PE150 HiSeq2500 data (FPR 0.00017% and sensitivity 96.60%), better than the PE50 data (FPR 0.0006% and sensitivity 94.15%). But for insertions and deletions (indels), we found lower accuracy for BGISEQ-500 data (FPR 0.00069% and 0.00067% for PE100 and PE50 respectively, sensitivity 88.52% and 70.93%) than the HiSeq2500 data (FPR 0.00032% and sensitivity 96.28%). Our dataset can serve as the reference dataset providing basic information not just for future development, but also for all research and applications based on the new sequencing platform.

Keywords: Genomics, sequencing, Next Generation Sequencing, BGISEQ-500

Data Description

Massively parallel sequencing technologies (also called as the second generation sequencing) generate large amount of data with lower cost, shorter reads and higher single base error rate comparing to Sanger sequencing technology[1]. With the large amount of data and well-developed analysis tools, second generation sequencing data can be used to effectively and accurately identify genomic variations[2]. Thus it has been widely applied in both research and application[3]. Currently there are several commercially available second generation sequencing platforms with differing performance and data features[4, 5]. With more and more research areas and applications to apply sequencing to, new sequencing platforms are being developed at a rapid pace. The BGISEQ-500 sequencer was first announced by BGI in October, 2015. It was developed based on the Complete Genomics™ sequencing technologies, and applied DNA NanoBalls (DNBs) technology[6] for sequencing library construction and combined primer anchor synthesis (cPAS) for sequencing. We present here a dataset generated from the BGISEQ-500 sequencer, including examples of the raw images and the final sequences. We also conducted variation calling using this dataset and compared the variation calling result to that from other sequencers. This dataset can be served as a useful reference for the community to develop bioinformatics methods and sequencing based applications on this new sequencing platform.

DNA preparation

NA12878 cell line (RRID:CVCL_7526) genomic DNA was ordered from the Coriell Institute, and contained 50 µg per tube. The genomic DNA was quantified by Qubit 3.0 fluorometer (Life Technologies, Paisley, UK) and the integrity was qualified on the 2% agarose gel to make sure the genomic DNA molecular was larger than 23kb and not substantially degraded.

Sequencing library preparation

For the sequencing library construction, the NA12878 genomic DNA was fragmented

by ultrasound on Covaris E220 (Covaris, Brighton, UK) to DNA fragments between 50 bp~800bp according to the manufacturer's instructions. The fragmented DNA was further selected to 100bp~300bp by AMPure XP beads (AGENCOURT). The selected DNA fragments were then repaired to obtain a blunt end and modified at 3'end to get a dATP as a sticky end. The dTTP tailed adapter sequence was ligated to both ends of the DNA fragments. The ligation product was then amplified for 8cycles and subjected to the following single strand circularization process. The PCR product was heat-denatured together with a special molecule which was reverse complemented to one special strand of the PCR product, and the single strand molecule was ligated using DNA ligase. The remaining linear molecule was digested with the exonuclease, finally obtaining a single strand circular DNA library (Figure 1a).

Sequencing

We conducted sequencing according to the BGISEQ-500 protocol (Figure 1b). There were three steps including making DNBs, loading DNBs and sequencing. For making DNBs, a 6 ng single strand circular DNA library was first PCR amplified for 10 minutes in an 80 μl reaction volume with pure water, buffer and DNB polymerase. After the PCR reaction, 20 μl DNBs stopping buffer was added to terminate the PCR reaction. Finally, we used the Qubit® ssDNA Assay Kit to quantify the DNBs on a Qubit® Fluorometer (concentration ≥10 ng/μL).

For loading DNBs, we first added 33 μl DNBs loading buffer to DNBs product from the last step, and the mixture was placed on the BGIDL-50 (the sample preparation machine). Then we selected the DNBs loading process (Version: sample load 2.0) to load DNB onto the sequencing chip, which included 96 minutes' loading time and 30 minutes' incubation at room temperature.

Finally, for sequencing, we followed to the BGISEQ-500 protocol. We selected sequence control software Version 1.1.0.10003, sequence process Version 1.0.06 and Zebracall process Version 0.5.0.13875 (the base calling software, and a detailed description can be found in the next section) for sequencing. Sequencing was initiated

after the sequencing reagents pre-loaded and sequencing chip installed, and this process was finished in ~72 hours.

Base calling and raw images

During sequencing, four channels of 16-bit grey scale images were captured by high resolution sCMOS with ~5.5 million pixels per image. About ~570K DNBs were loaded onto the grid-patterned arrays of spots which were photolithographically etched and surface modified on the sequencing chip. The spots were illuminated by the lasers with different wavelengths. Intensity from neighboring channel would also be observed due to crosstalk effect. The sequences of DNBs were base-called by the software Zebra call (base calling software developed for BGISEQ-500). After background subtraction and registration of images from 4 channels, intensities of DNBs were extracted according to a template of grid-pattern. Correction within channels and neighbor cycles was applied to increase the quality and stabilization. The cross-talk between intensities from the four fluorophores is caused by the imperfect wavelength filtering of optical filters isolating the bands of wavelength from the four types of fluorophore molecules. A regression technique can identify correlations in our intensity data and correct for them. For example, in order to correct the crosstalk between 2 channels (C and G), the correction of the C background intensity can be found by linear regression after eliminating DNBs that do have true signal in the C channel. Such DNBs can be identified by searching for DNBs that have the C intensity as the maximum of the four intensities and, to retain just DNBs that are not too dim or noisy, we take only DNBs that have less than 80% of the C intensity for the remaining three other intensities. This leaves us with reasonably well performing DNBs that most likely do not contain C at the currently interrogated position. Linear regression was then carried out for these background G intensities as a function of the C intensities. All of the G intensities can in turn be corrected for this cross-talk from the C channel by subtracting from them the expected background intensity produced by a given C intensity. Such regression can be done for each

channel and simultaneously correcting for all of the correlations outlined above using a multiple linear regression. After all correction steps, the base with highest probability will be called according to the scale of intensities. When the whole sequencing was finished, the binary file with bases and quality score were converted into FASTQ format with Phred+33 quality score.

In order to map the base call quality to Phred+33 score, a prior probability model was constructed by the scale of intensities from channels. Bases were separated to 10404 groups according to different parameters which may affect the confidence level of base calling. The base-calling error probabilities(P) of each group was calculated by the mismatch distribution from repeated sequencing of the standard reference genome. Quality scores (Q) were calculated by the definition of Phred+33 quality scores:

$$Q = -10\ log_{10}P$$

A huge table was constructed and hard coded into the base call program to look up a corresponding quality score by different parameters.

An example dataset of the images was included and the base calling process was illustrated in Figure 2.

Results

Sequencing data summary

The sequencing data consists of four lanes, with two of PE100 and the other two of PE50 (Table 1). First, we analyzed the sequencing quality by identifying the low quality reads. Although previous studies revealed that raw data filtering would not substantially affect variation calling result [7, 8], we found slight different performances of variation calling using different raw data filtering criteria (Table S1). Thus, we determined low quality reads as reads which had more than 10% bases with sequencing quality lower than 10, and reads which had more than 1% Ns (ambiguous bases). In this way, we identified 11.9% (9.2% low quality reads and 2.7% ambiguous reads) low quality reads in PE100 data and 12.3% low quality raw reads in PE50 data

(5.4% low quality reads and 6.9% ambiguous reads). In order for comparison, we selected similar amount of data (8 sequencing libraries and 16 lanes, PE150 reads, ~98.5 G bp data) from a public Illumina HiSeq2500 dataset of this cell line generated by GIAB (Genome in a Bottle)[9]. Using the same criteria for low quality identification, we identified 7.95% low quality reads (7.7% low quality reads and 0.25% ambiguous reads). Excluding these low quality reads, we then further analyzed the reads quality by plotting the distributions of base quality scores and GC content against those of the HiSeq2500 data (Figure 3). Thus we found higher proportion of low quality reads, more stable base quality distribution along the reads (Figure 3 a-b) and lower overall single base quality scores (Figure 3 c). And we observed some secondary peak in the GC content distribution of BGISEQ-500 data, indicating higher GC bias (Figure 3 d).

Variation calling and false positive/negative ratios estimation

In order to further depict the data quality and test applications of the new sequencing platform, we carried out variation calling using this dataset. We adapted the widely used pipeline (BWA[10] and GATK[11-13], an illustration of the pipeline and key parameters can be found in Figure 4a) for variation calling. We observed higher mapping rate, similar sequencing coverage and similar sequencing uniformity of the two BGISEQ-500 datasets compared to the HiSeq2500 dataset (Table 2). The lower unique mapping rate probably reflected the shorter read length of the dataset (2×50 bp and 2×100 bp comparing to 2×150 bp). We also observed slightly higher duplication rate and comparable mismatch rate in the BGISEQ-500 PE100 dataset comparing to the HiSeq2500 data (Table 2).

In total, we identified ~3.4 million SNPs using the BGISEQ-500 datasets (3.45 million for PE50 data and 3.48 million for PE100 data), more than 3.6 million SNPs identified using HiSeq2500 data (Table 3). While for indels (insertion and deletions), we identified 842,058 from BGISEQ-500 PE100 data, comparing to 553,842 identified from BGISEQ-500 PE50 data. Using the HiSeq2500 data, we identified

733,797 indels. The SNPs identified using BGISEQ-500 datasets were similar to those identified from HiSeq2500 data in different features including dbSNP rate, proportion of SNPs in different regions related to genes and Ti/Tv (transition/transversion) ratio, which indirectly reflected the SNP accuracy. We also observed similar situation for indels.

Further to assess the accuracy of the variations, we used the high confident variations previously identified in NA12878 provided by GIAB (Genome in A Bottle)[14]. Using the methods provided by GIAB, we estimated the false positive rates and sensitivity for BGISEQ-500 PE50 and PE100 data compared to those of HiSeq2500 data (Table4). The SNP sensitivity was lower for the BGISEQ-500 datasets (96.20% for PE100 and 94.15% for PE50) than HiSeq2500 data (96.60%). And the SNP false positive rate (FPR) was similar for the BGISEQ-500 PE100 data (0.00020%) compared to HiSeq2500 data (0.00017%), and lower than the BGISEQ-500 PE50 data (0.0006%). For indels, BGISEQ-500 PE100 data resulted in worse performance with lower sensitivity (88.52%) than the HiSeq2500 PE150 data with sensitivity of 96.28%. In contrast, HiSeq2500 PE150 data shows lower FPR (0.00032%) than BGISEQ-500 PE100 data (0.00069%). The BGISEQ-500 PE50 data resulted in sensitivity of 70.93% and FPR of 0.00067%. The difference performances of indel calling might also be caused by read length difference (50 or 100 bp comparing to 150 bp), in addition to sequencing quality, mapping accuracy, etc.

Furthermore, to depict variation calling accuracy in different genomic regions, we compared the false negative rate (FNR), FPR and sensitivity in different genome context given by GIAB (Figure S1). For the coding sequences, data from the two platforms have similar FNR, FPR and sensitivity (3.85% *vs.* 2.52%, 0.00012% *vs.* 0.00015% and 96.15% *vs.* 97.48% accordingly). For the regions which are difficult to sequence, including some of the promoters [15], substantially high GC content (>55%) regions, substantially low GC content (<30%) regions, regions with multiple variations (more than 1 variations within 50 bp), regions with compound variations,

repeats and segmental duplications, BGISEQ-500 data has a higher FNR, lower sensitivity and lower FPR (Figure S1).

Discussion

Using the new sequencer, BGISEQ-500, we obtained one run of PE50 data and the other run of PE100 data. The raw data were ~135.5 Gbp and ~153.6 Gbp respectively, and were generated from two chips (~72 hours). Thus the sequencing throughput and turnaround time were comparable to HiSeq2500 sequencer Rapid mode v1 (~80 Gbp per single flow cell and ~40 hours). Both the single base quality and read quality (reflected by duplication rate, mapping rate and unique mapping rate) were basically comparable to those of the HiSeq2500 data. Furthermore, the variation calling result was similar to that identified using similar amounts of HiSeq2500 data, further reflecting that the sequencer can be used in different research and applications. With Future improvements over data quality, sequencing length, different and optimized insert sizes of the paired reads, as well as specially modified or designed software/bioinformatics tools, the performance can be further improved. In the meantime, quality of the whole genome sequencing data also reflected feasibility of applying this sequencing platform for other sequencing purposes including transcriptome, epigenome, metagenome etc. From this first reference dataset of sequencing data from BGISEQ-500 sequencer, we provided an overview and some basic information for the new sequencing platform. This dataset can serve as reference for all the research using the BGISEQ-500 sequencing platform. And we anticipate it to help stimulating the further technical improvement and development of novel tools for accurately analyzing this data.

Availability of supporting data

The BGISEQ-500 sequences described in this article are available in the GigaDB repository (PE 50[16] and PE 100 [17]), and the European Nucleotide Archive under accession number ERP017158. This GigaDB entry also contains examples of the raw image data including images of all the sequencing cycles in a small region and images of the first and last 10 cycles of the whole flowcell [16]. Future data will also be updated via the GigaDB repository with versions indicated.

Abbreviations

bp: base-pair; DNBs: DNA nanoballs; FNR: false negative rate; FPR: false positive rate; GIAB: Genome in A Bottle; PE50: pair-end 50 bp; PE100: pair-end 100 bp; SNPs: Single Nucleotide Polymorphisms; indels: insertions and deletions

Competing interests

JH, YX, SQ, XM, HC, TY, NS, ZY and SG are involved in the beta test of the BGISEQ-500 sequencer. X Liang, CG, YL, HL, HJ, X Liu and FM are involved in the BGISEQ-500 sequencer development, library construction technology optimization, base calling software development, or alpha and beta tests.

Authors' contributions

JH, ZY, FM and SG designed the project. YX, SQ and CG conducted sample preparation and sequencing library construction. HL, XM, HC, TY and NS conducted sequencing. X Liang, JR, JW, YL, X Liu, HJ, JR, JW, WZ, YC and SL conducted data analysis. X Liu, X Liang, YL, CG, HL, JH and HJ wrote the manuscript.

Acknowledgements

This work was supported by following funding: the National High Technology Research and Development Program ("863" Program) of China (Project No.

2011AA02A115), the Technology Innovation and Developing Plan of Shenzhen (Project No. CXZZ20140904154910774).

Author details

[1] National Institutes for food and drug Control (NIFDC), Beijing 100050, P. R. China

[2] BGI-Shenzhen, Shenzhen 518083, P. R. China

[3] State Food and Drug Administration Hubei Center for Medical Equipment Quality Supervision and Testing, Wuhan 430000, P. R. China

[4]BGI-Qingdao, Qingdao 266555, China

References

1. Metzker ML: **Sequencing technologies - the next generation**. *Nat Rev Genet* 2010, **11**(1):31-46.
2. Wang J, Wang W, Li R, Li Y, Tian G, Goodman L, Fan W, Zhang J, Li J, Guo Y *et al*: **The diploid genome sequence of an Asian individual**. *Nature* 2008, **456**(7218):60-65.
3. Goodwin S, McPherson JD, McCombie WR: **Coming of age: ten years of next-generation sequencing technologies**. *Nat Rev Genet* 2016, **17**(6):333-351.
4. Mardis ER: **Next-generation sequencing platforms**. *Annu Rev Anal Chem (Palo Alto Calif)* 2013, **6**:287-303.
5. Quail MA, Smith M, Coupland P, Otto TD, Harris SR, Connor TR, Bertoni A, Swerdlow HP, Gu Y: **A tale of three next generation sequencing platforms: comparison of Ion Torrent, Pacific Biosciences and Illumina MiSeq sequencers**. *BMC Genomics* 2012, **13**:341.
6. Drmanac R, Sparks AB, Callow MJ, Halpern AL, Burns NL, Kermani BG, Carnevali P, Nazarenko I, Nilsen GB, Yeung G *et al*: **Human genome sequencing using unchained base reads on self-assembling DNA nanoarrays**. *Science* 2010, **327**(5961):78-81.
7. Hwang S, Kim E, Lee I, Marcotte EM: **Systematic comparison of variant calling pipelines using gold standard personal exome variants**. *Scientific reports* 2015, **5**:17875.
8. Liu Q, Guo Y, Li J, Long J, Zhang B, Shyr Y: **Steps to ensure accuracy in genotype and SNP calling from Illumina sequencing data**. *BMC genomics* 2012, **13 Suppl 8**:S8.
9. Zook JM, Catoe D, McDaniel J, Vang L, Spies N, Sidow A, Weng Z, Liu Y, Mason CE, Alexander N *et al*: **Extensive sequencing of seven human genomes to characterize benchmark reference materials**. *Sci Data* 2016, **3**:160025.
10. Li H, Durbin R: **Fast and accurate short read alignment with Burrows-Wheeler transform**. *Bioinformatics* 2009, **25**(14):1754-1760.
11. McKenna A, Hanna M, Banks E, Sivachenko A, Cibulskis K, Kernytsky A, Garimella K, Altshuler D, Gabriel S, Daly M *et al*: **The Genome Analysis Toolkit: a MapReduce framework for analyzing next-generation DNA sequencing data**. *Genome Res* 2010, **20**(9):1297-1303.
12. DePristo MA, Banks E, Poplin R, Garimella KV, Maguire JR, Hartl C, Philippakis AA, del Angel G, Rivas MA, Hanna M *et al*: **A framework for variation discovery and genotyping using next-generation DNA sequencing data**. *Nat Genet* 2011, **43**(5):491-498.
13. Van der Auwera GA, Carneiro MO, Hartl C, Poplin R, Del Angel G, Levy-Moonshine A, Jordan T, Shakir K, Roazen D, Thibault J *et al*: **From FastQ data to high confidence variant calls: the Genome Analysis Toolkit**

best practices pipeline. *Curr Protoc Bioinformatics* 2013, **43**:11 10 11-33.

14. Zook JM, Chapman B, Wang J, Mittelman D, Hofmann O, Hide W, Salit M: **Integrating human sequence data sets provides a resource of benchmark SNP and indel genotype calls**. *Nat Biotechnol* 2014, **32**(3):246-251.
15. Ross MG, Russ C, Costello M, Hollinger A, Lennon NJ, Hegarty R, Nusbaum C, Jaffe DB. **Characterizing and measuring bias in sequence data.** Genome Biol. 2013 May 29;14(5):R51.
16. Huang, J; Liang, X; Xuan, Y; Geng, C; Li, Y; Lu, H; Qu, S; Mei, X; Chen, H; Yu, T; Sun, N; Rao, J; Wang, J; Zhang, W; Chen, Y; Liao, S; Jiang, H; Liu, X; Yang, Z; Mu, F; Gao, S (2016): **BGISEQ-500 sequencer first reference dataset** GigaScience Database. http://dx.doi.org/10.5524/100252
17. Huang, J; Liang, X; Xuan, Y; Geng, C; Li, Y; Lu, H; Qu, S; Mei, X; Chen, H; Yu, T; Sun, N; Jiang, H; Liu, X; Yang, Z; Mu, F; Gao, S (2017): **An updated reference human genome dataset of the BGISEQ-500 sequencer** GigaScience Database. http://dx.doi.org/10.5524/100274
18. Andrews S: **FastQC, a quality control tool for high throughput sequence data**. 2016.
19. **Genome in a Bottle Consortium high-confidence VCF and BED datasets** ftp://ftp-trace.ncbi.nlm.nih.gov/giab/ftp/release/NA12878_HG001/NISTv3.3/NA12878_GIAB_highconf_CG-IllFB-IllGATKHC-Ion-Solid-10X_CHROM1-X_v3.3_highconf.bed

Figure Legends

Figure 1. Flowchart of library construction and sequencing. The library construction includes fragmentation, size selection, end-repair and A-tailing, adaptor ligation and PCR amplification and splint circularization (**a**). The sequencing includes making DNBs, loading DNBs and sequencing (**b**).

Figure 2. Raw image data processing on the BGISEQ-500 platform. a. Registration of images from different channels. Relative coordinates will be calculated according to the pattern layout of DNBs. **b. Intensity correction between channels and cycles.** Correction of the optical and chemical interferences on different channels and the neighbor cycles was applied. **c. Connecting called bases to FASTQ.** Bases from all cycles will be collected and converted to FASTQ format. Phred score calculation and statistics will be applied during the conversion.

Figure 3. Quality control of the dataset after data filtering. Base-wise quality score distributions of the first read (**a**, from left to right, BGISEQ-500 PE50, BGISEQ-500 PE100 and HiSeq2500 PE150) and the second read (**b**, from left to right, BGISEQ-500 PE50, BGISEQ-500 PE100 and HiSeq2500 PE150)**.** For each position along the reads, the quality scores of all reads were used to calculate the mean, median and quantile values thus the box plot can be shown. The overall quality score distribution of BGISEQ-500 and HiSeq2500 data (**c**). GC content distribution of the BGISEQ-500 and HiSeq2500 data (**d**). FastQC [18] was used for the calculation.

Figure 4. Variation calling based on the dataset. The major steps included data filtering, alignment and variation calling, and the major parameters are also indicated.

Tables

Table 1. Summary of the dataset*.

Sequencing Type	Read (×10^6)	Bases (Gbp)	GC content	>Q20	>Q30
PE50	2,379	118.94	41.62%	96.00%	87.02%
PE100	1,159	115.88	41.28%	96.39%	87.13%

*This dataset was from two runs of the BGISEQ-500 sequencer (PE50 and PE100). '>Q20/Q30 percentage' indicates the percent of bases with quality score (-10×lg(error rate)) higher than 20 and 30 (indicating error rates of 1% and 1‰ respectively).

Table 2. Mapping statistics of the dataset*.

Metrics	BGISEQ-500 PE50	BGISEQ-500 PE100	HiSeq2500 PE150
Clean reads	2,378,725,921	1,136,008,901	708,941,148
Clean bases (bp)	118,936,296,050	113,600,890,100	104,923,289,904
Mapping rate	97.87%	99.22%	99.05%
Unique rate	93.17%	96.47%	97.06%
Duplicate rate	6.26%	2.47%	1.52%
Mismatch rate	0.34%	0.58%	0.56%
Average sequencing depth	37.57	37.44	34.52
Coverage	99.28%	99.12%	99.06%
Coverage at least 4×	98.90%	98.69%	98.60%
Coverage at least 10×	97.97%	97.81%	97.83%
Coverage at least 20×	95.78%	96.06%	94.81%

*The statistics shown here are calculated based on the clean reads (raw reads after filtering, the two platforms' data went through the same filtering process). Unique mapping rate indicates proportion of reads with unique alignment in the genome.

Table 3. Variation statistics of the dataset*.

	BGISEQ-500 PE50	BGISEQ-500 PE100	HiSeq2500 PE150
SNPs	3,451,124	3,477,642	3,609,606
1000genome and dbSNP	3,242,083	3,288,653	3,347,441
1000genome specific	1,260	420	693
dbSNP specific	180,935	179,967	243,256
dbSNP rate	99.19%	99.74%	99.48%
Novel	26,846	8,602	18,216
Homozygous	1,426,328	1,433,490	1,472,063
Heterzygous	2,024,796	2,044,152	2,137,543
Synonymous	19,880	20,012	20,860
Ti/Tv	2.0462	2.065	2.0427
dbSNP Ti/Tv	2.0608	2.0693	2.0503
Novel Ti/Tv	0.8948	0.9775	1.0544
Indels	553,842	842,058	733,797
1000genome and dbSNP	260,157	320,741	314,161
1000genome specific	7,007	22,919	20,049
dbSNP specific	211,846	326,984	285,834
dbSNP rate	85.22%	76.92%	81.77%
Novel	74,832	171,414	113,753
Homozygous	206,163	295,492	300,013
Heterzygous	347,679	546,566	433,784

*1000genome and dbSNP equals the number of SNPs that are found in both 1000 genome and dbSNP databases (version 147 was used), 1000genome specific equals the number of SNPs that are only found in 1000 genomes database. dbSNP rate equals the number of SNPs found in dbSNP database/total detected SNPs. Novel SNP equals the number of SNPs that are

not found in SNP database. Ti/Tv equals the ratio of SNP type are transition/SNP type are transversion.

Table 4. Performances of variation calling of dataset*.

Variant type	Metrics	BGISEQ-500 PE50	BGISEQ-500 PE100	HiSeq2500 PE150
SNPs	True Positive	3,006,132	3,071,579	3,084,449
	False Positive	15,203	6,907	4,318
	False Negative	186,825	121,379	108,508
	Precision	99.50%	99.78%	99.86%
	Sensitivity	94.15%	96.20%	96.60%
	FPR	0.00060%	0.00020%	0.00017%
	FNR	5.85%	3.80%	3.40%
indels	True Positive	261,867	326,810	355,728
	False Positive	16,931	22,246	7,981
	False Negative	107,311	42,391	13,751
	Precision	93.93%	93.63%	97.81%
	Sensitivity	70.93%	88.52%	96.28%
	FPR	0.00067%	0.00069%	0.00032%
	FNR	29.7%	11.48%	3.72%

*Above first four metrics are calculated by using rtg-tools software. True Positive (TP) is the number of SNPs that are found in high-confidence reference dataset, False Positive (FP) is the number of SNPs that are not found in reference dataset, False Negative (FN) is the number of SNPs that are found in high-confidence reference dataset but are not found in reference dataset. Precision is TP/(TP+FP)*100. Sensitivity is TP/(TP+FN)*100. FPR is FP/(all high-confident region length-TP-FN)*100, where all high-confident region length equals 252,9164,928bp that comes from GIAB released high confidence variants datasets [19]. FNR is FN/(FN+TP)*100.

Figure 1

Click here to download Figure Figure 1.pdf

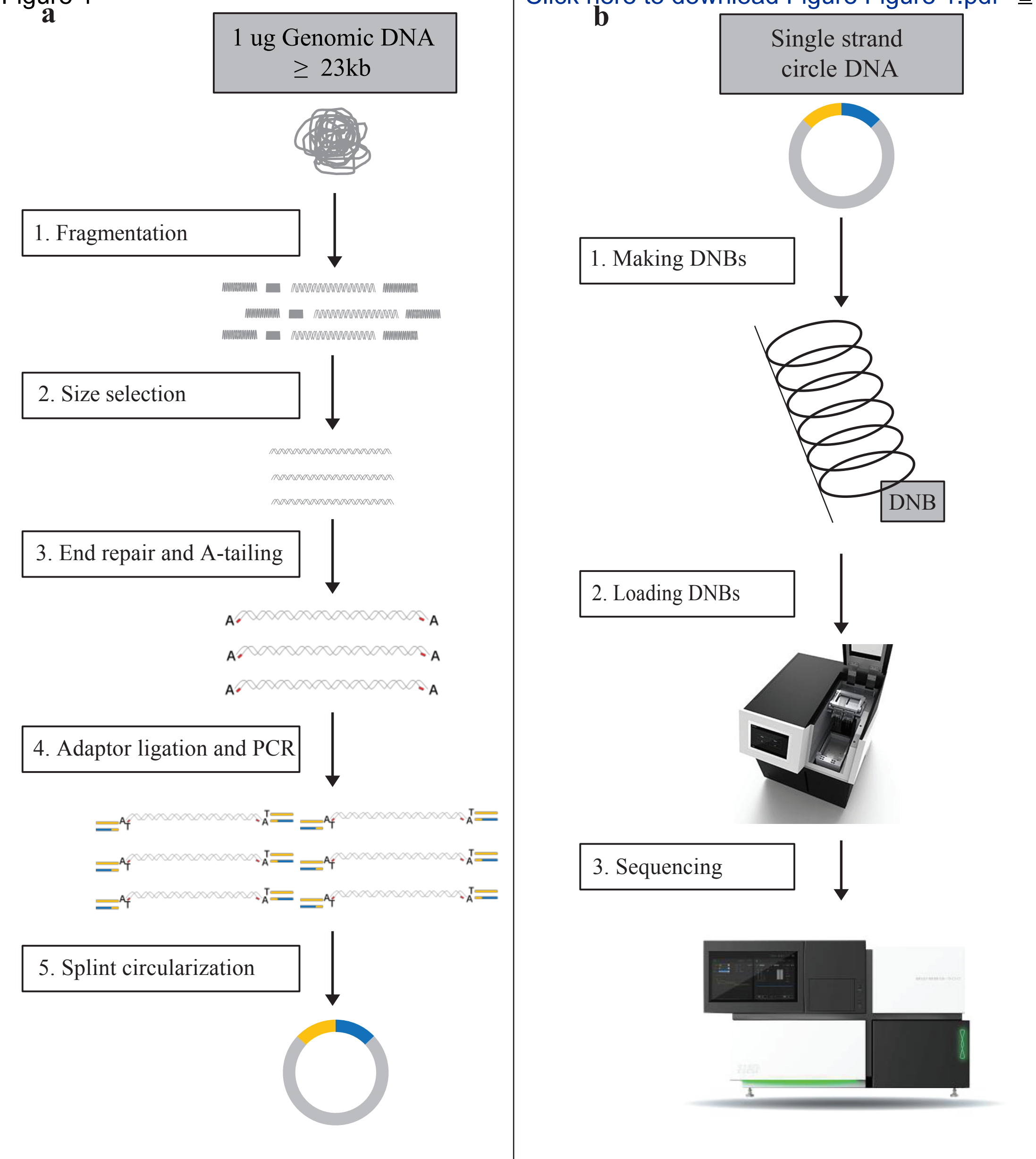

Figure 2
Click here to download Figure Figure 2.pdf

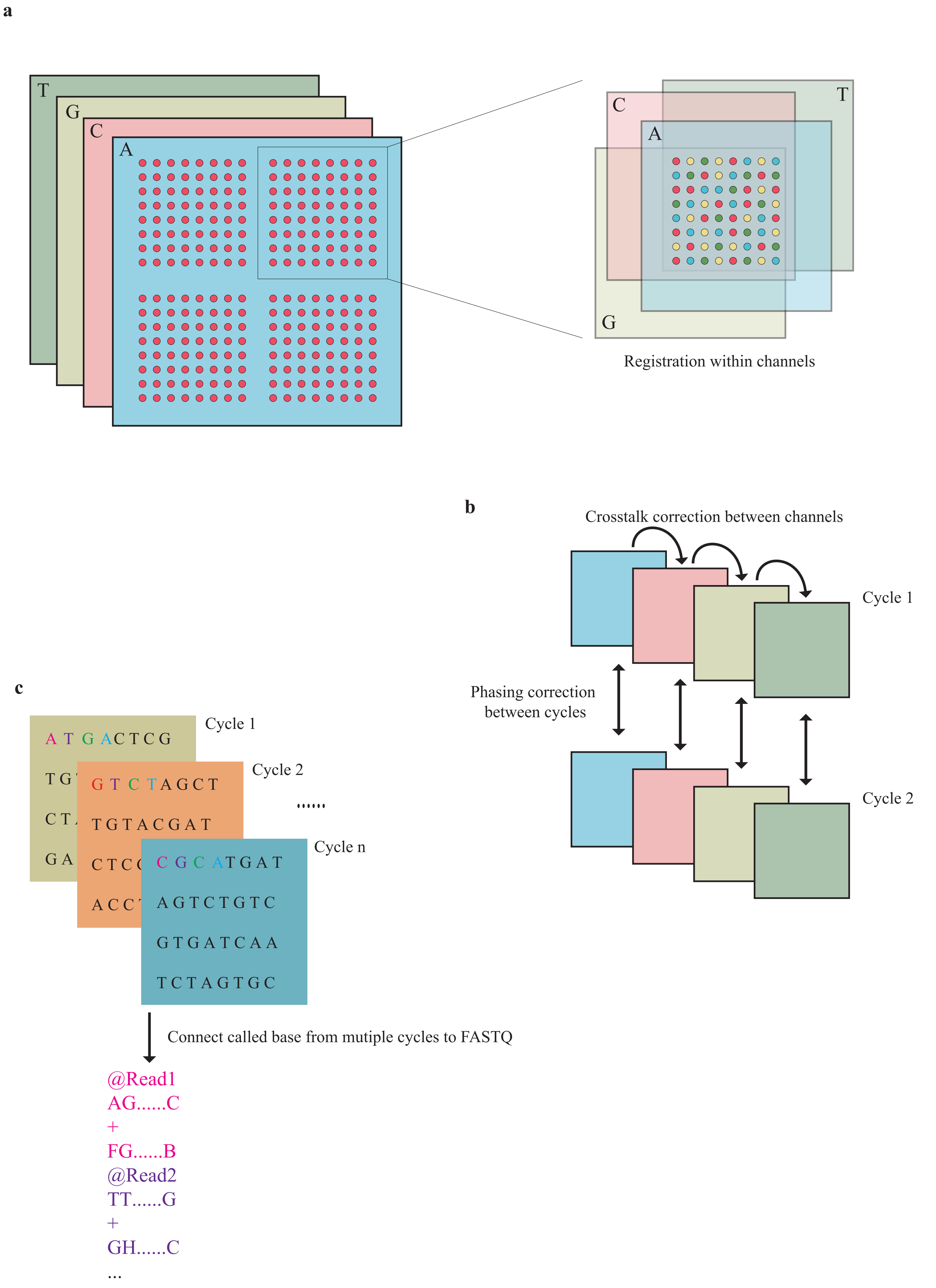

Figure 3

Click here to download Figure Figure 3.pdf

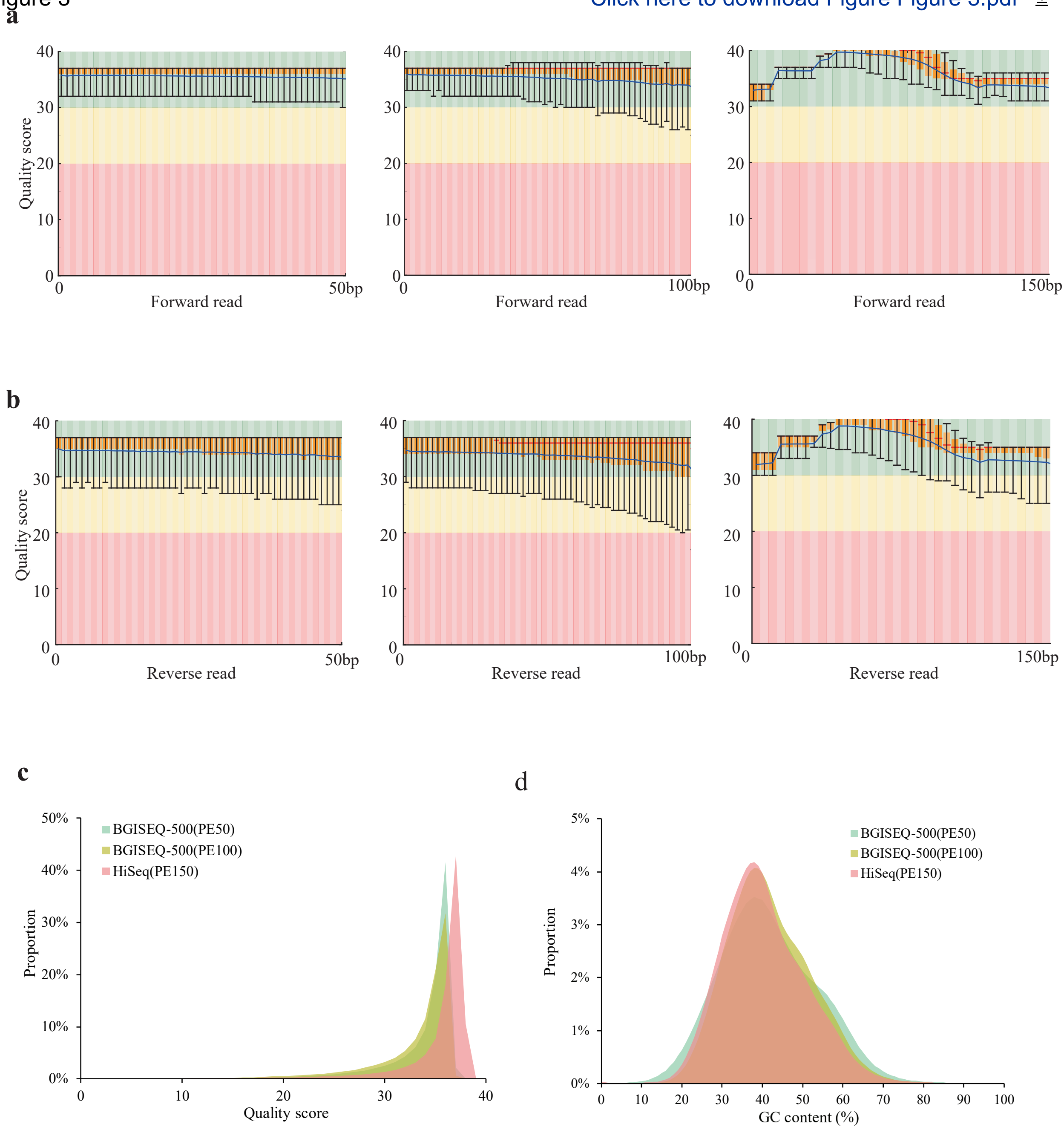

Figure 4 Click here to download Figure Figure 4.pdf

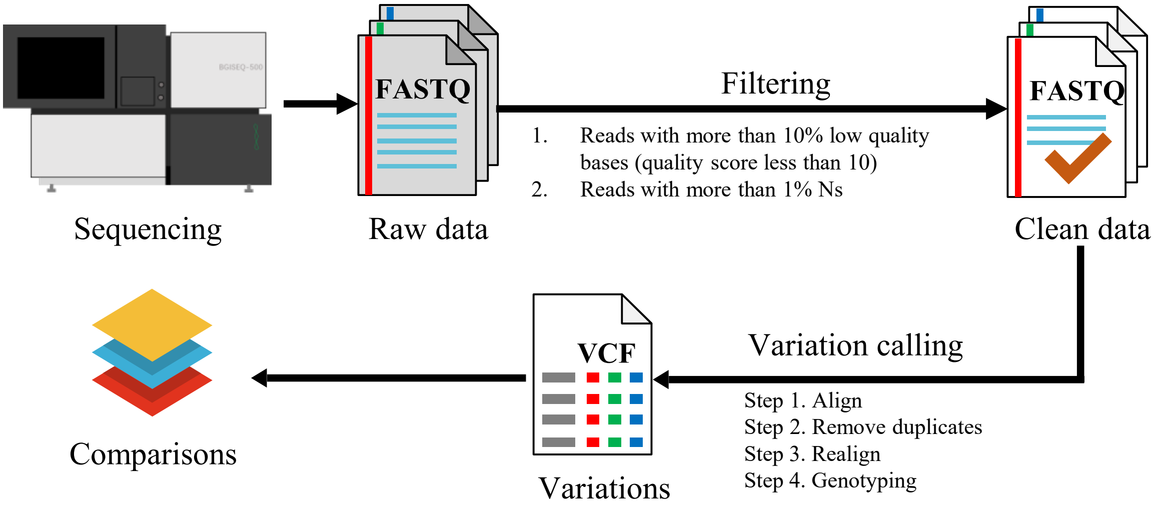

Click here to access/download
Supplementary Material
Supplementary meterials.docx

Point-to-point response

Click here to access/download
Supplementary Material
RebutallLetter.docx

Personal Cover Click here to download Personal Cover CoverLetter.docx

Dear Editor,

Thanks for potentially accepting our manuscript entitled *A reference human genome dataset of the BGISEQ-500 sequencer* for possible publication as a datanote in *GigaScience*.

We have revised our manuscript again according to the suggestions of the reviewer #2. During the revision, we revised the mistakes in grammar and sentences as pointed out by the reviewer, and we also added the variation calling performance assessment of different data filtering criteria to the supplementary material according to the reviewer's advice. Thus we have addressed all the questions raised by Reviewer #2 as you can find out in the rebuttal letter with point-to-point response letter.

The revised manuscript has ~2,400 words in the main text (including the abstract), with four figures and four tables, and one supplementary file (with detailed parameters for the analysis, the accuracy assessment in different genome content as well as performances of two platforms under different filtering threshold). Please let us know if other information needed, and we are looking forward to your response.

Xin Liu,

BGI Research
