# Supplementary material for: A reference human genome dataset of the BGISEQ-500 sequencer
Source: Gigascience. 2017 Apr 1;6(5):1–9. doi: 10.1093/gigascience/gix024 (PMC5467036; doi:10.1093/gigascience/gix024)
Supplement: Supplemental material — Additional file 1: Figure S1: Single nucleotide polymorphism stratification performance between BGISEQ-500 and HiSeq2500. We used stratification files from the Global Alliance for Genomics and Health (GA4GH) Benchmarking Team and the Genome in a Bottle Consortium that were intended as a standard resource of bed files for use in stratifying true positive, false positive, and false negative variant calls into different categories. We list detailed information about different regions. Additional file 1: Table S1: Performances of variation calling under different filtering threshold conditions. [file gix024_Supp.docx]

1. **Options of program and software in each analysis steps.**

- Filtering

SOAPnuke 1.5.3: SOAPnuke filter -l 10 -q 0.1 -n 0.01 -Q 2 -G -f AAGTCGGAGGCCAAGCGGTCTTAGGAAGACAA -r CAACTCCTTGGCTCACAGAACGACATGGCTACGATCCGACTT

- Alignment and genotype
  1. GAEA pipeline forBGISEQ-500 PE50 data
- Alignment

bwa 0.7.10: bwa alnpe -t 6 -i 10 -q 10 hg19.fa

- BamSort

-ref <reference file>

-o <output directory>

-F <output file format, 0 is CRAM, 1 is SAM, 2 is BAM>

- Remove duplication.

-I <input alignment file> -O <output directory> -I <input file type, 0 is BAM, 1 is SAM, default: 0>

- Realignment

--align <input rmdup alignment file>

--out <output directory> --ref <reference file>

- Baserecal, which is consists of two steps: reacal and printreads
  1. Recal

--ref <reference file>

--knowSites <known SNP file, e.g. dbSNP VCF file>

- 1. Printreads

-i <input realignment file>

-o <output directory>

-f <reference file>

-b <recal step result>

- Genotype

-genotype_likelihoods_model <calculation model, SNP/INDEL/BOTH>

-stand_call_conf <The minimum phred-scaled confidence threshold at which variants should be called, default is 30.0>

-stand_emit_conf <The minimum phred-scaled confidence threshold at which variants should be emitted (and filtered with LowQual if less than the calling threshold), default is 30.0>

-dbsnp < known dbSNP VCF file >

- 1. BWA + GATK pipeline forHiSeq2500 PE150 and BGISEQ-500 PE100 data
- Alignment

BWA v0.7.13: bwa mem -t 8 -M -Y <reference.fa> <read1.fq.gz> <read2.fq.gz>

- BamSort

samtools v1.3:samtools sort -m 1000000000 -T <output temporary files prefix> <output file name>

- MarkDuplication

picard-tools v1.119: java -jar MarkDuplicates.jar

I=<input sorted bam>

O=<output rmdup bam>

M=<output duplication metrics file>

REMOVE_DUPLICATES=false

- BAMindex

samtools v1.3: samtools index <input rmdup bam>

- Realignment

GATK v3.3.0: java -jar GenomeAnalysisTK.jar

-T RealignerTargetCreator

-R <reference.fa>

-I <input sorted rmdup bam>

-o <output intervals file>

-known <Mills_and_1000G_gold_standard.indels.hg19.vcf>

-known <1000G_phase1.indels.hg19.vcf>

java -jar GenomeAnalysisTK.jar

-T IndelRealigner

-R <reference.fa>

-I <input sorted rmdup bam>

-targetIntervals <output intervals file>

-o <output realignment bam>

-known <Mills_and_1000G_gold_standard.indels.hg19.vcf>

-known <1000G_phase1.indels.hg19.vcf>

- BaseRecalibrator(BQSR)

java -jar GenomeAnalysisTK.jar

-T BaseRecalibrator

-nct 8

-R <reference.fa>

-I <input realignment bam>

-o <output recalibration table file>

-knownSites Mills_and_1000G_gold_standard.indels.hg19.vcf

-knownSites 1000G_phase1.indels.hg19.vcf

-knowSites hg19_snp147.txt

java -jar GenomeAnalysisTK.jar

-T PrintReads

-R <reference.fa>

-I <input realignment bam>

-BQSR <input recalibration table file>

-baqGOP 30

-o <output BQSR bam>

- Reduce bam

java -jar GenomeAnalysisTK.jar

-T ReduceReads

-R <reference.fa>

-I <input BQSR bam>

-o <output final bam>

- Genotype

java -jar GenomeAnalysisTK.jar

-T HaplotypeCaller

-R <reference.fa>

-I <input final bam>

--dbsnp dbsnp_135.hg19.modify.vcf

-stand_call_conf 30.0 -stand_emit_conf 10.0

-o <raw snp indel vcf>

- Variants validation

rtg-tools v3.7: rtg vcfeval

-b < baseline variants vcf>

-c <called variants vcf>

-e <evaluate within regions contained in the supplied BED file>

-t < SDF of the reference genome the variants are called against>

-o < output directory>


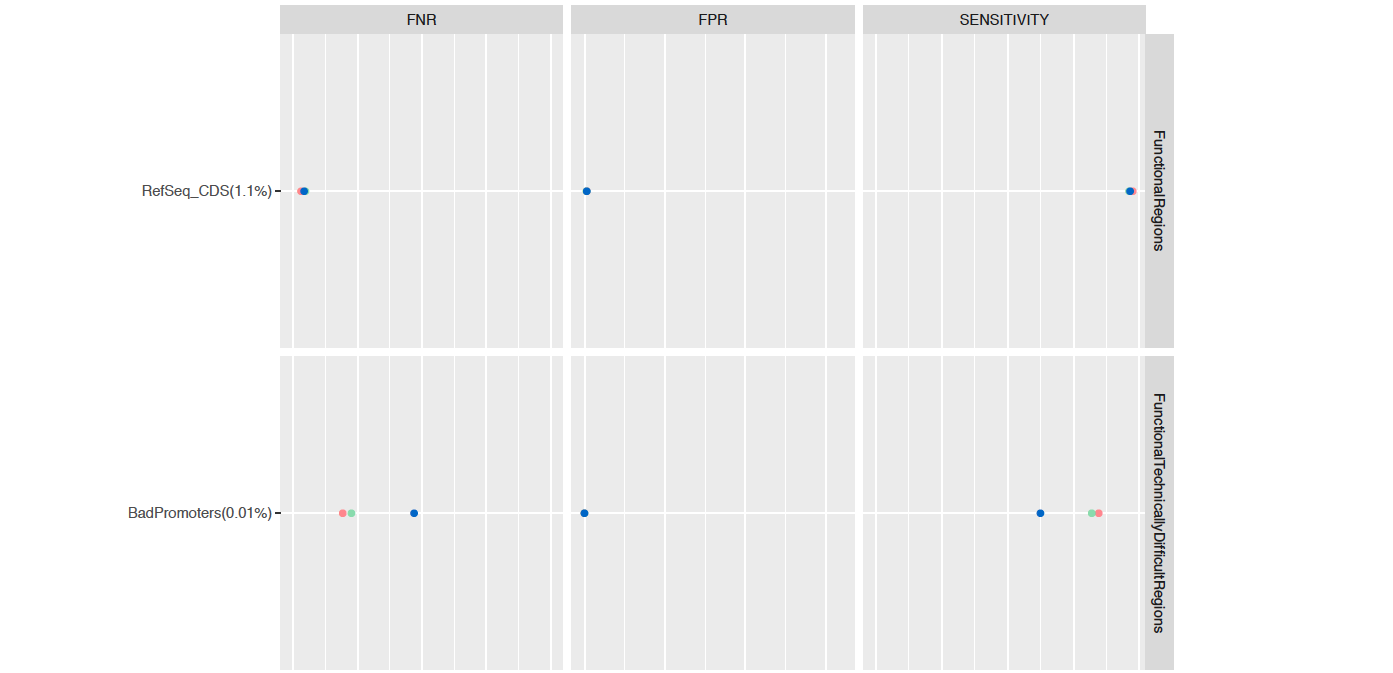


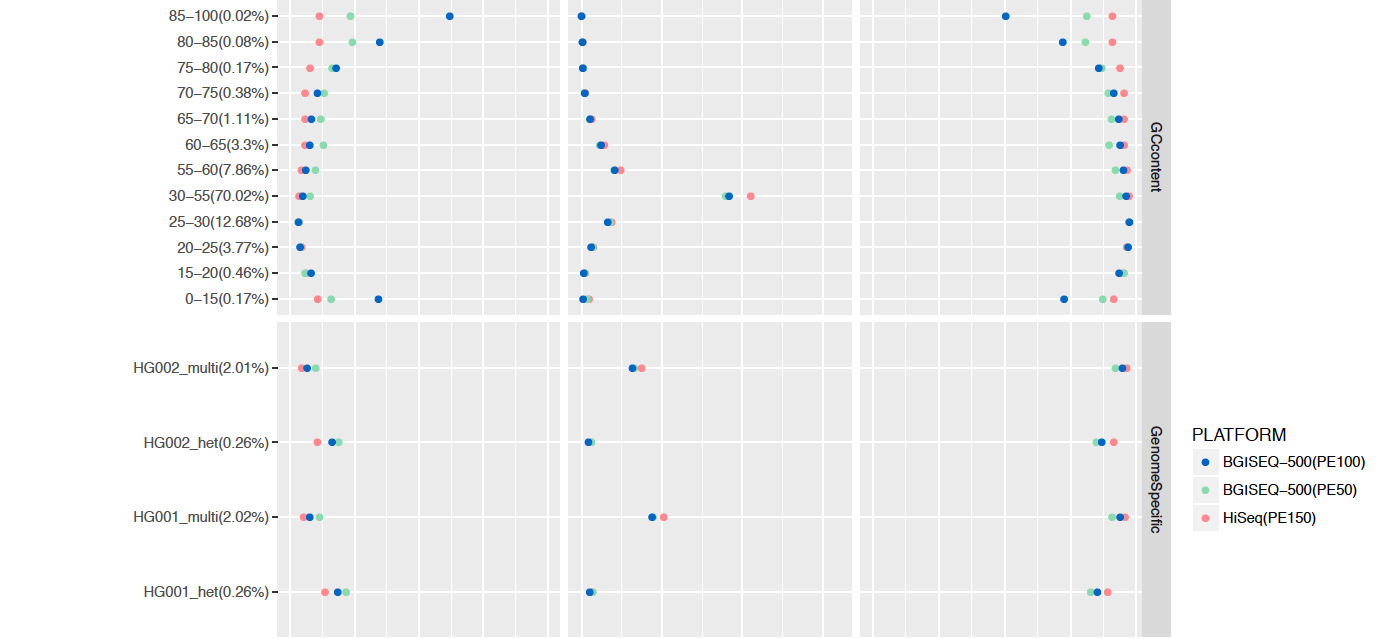


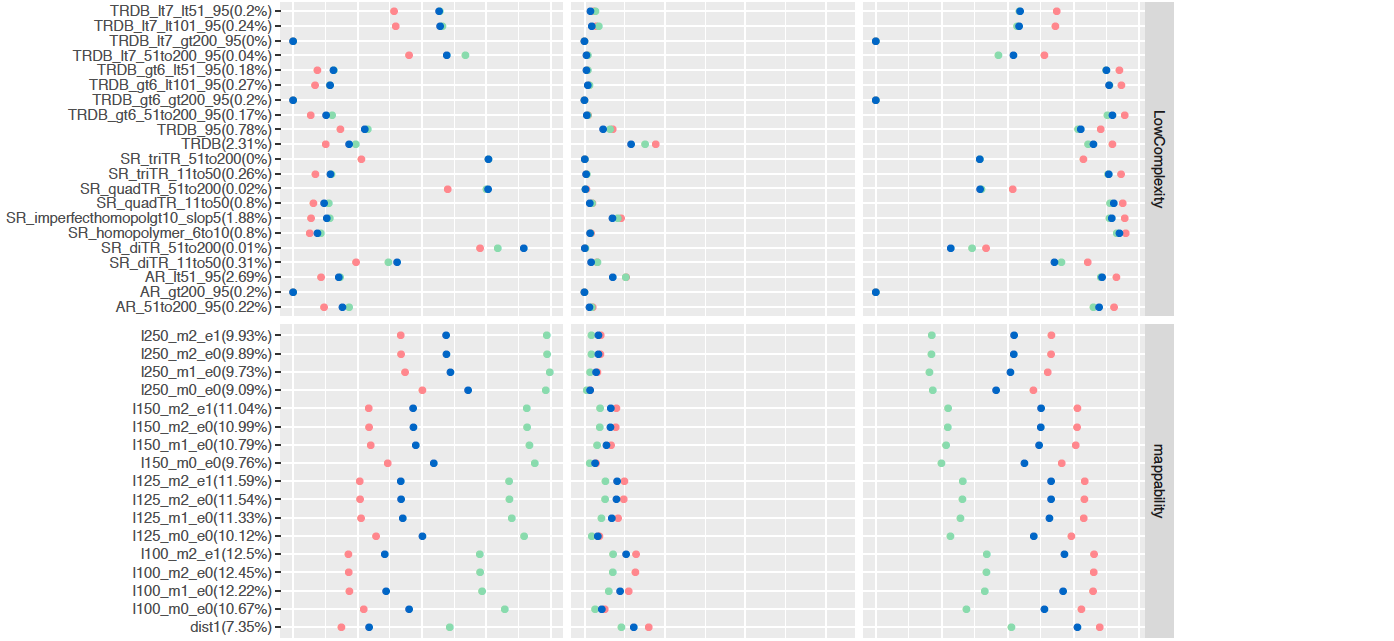


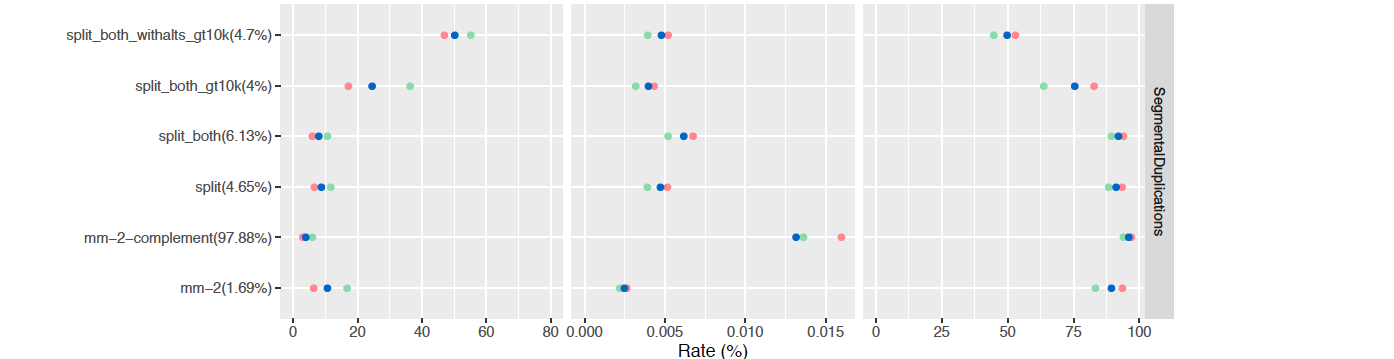


**Figure S1. SNP Stratification performance between BGISEQ-500 and HiSeq2500.** We used Stratification files from the Global Alliance for Genomics and Health (GA4GH) Benchmarking Team and the Genome in a Bottle Consortium, which intended as standard resource of bed files for use in stratifying true positive, false positive, and false negative variant calls into different categories. Following are detailed information about different region:

1. FunctionalRegions

RefSeq_CDS:the union of RefSeq coding sequences with only 1-22, X, Y and MT.

1. FunctionalTechnicallyDifficultRegions

BadPromoters

difficult-to-sequence promoters, which means the 1,000 transcription start sites or first exons with the lowest relative coverage based on low coverage by an Illumina data set. <http://genomebiology.com/2013/14/5/R51> (doi:10.1186/gb-2013-14-5-r51).

1. GCcontent

0-15,15-20,20-25,25-30,30-55…

Genome regions within different GC contents, such as 0-15 means regions with 0% ~ 15%. <https://groups.google.com/d/msgid/ga4gh-dwg-benchmarking/CAPipXk%2B4PCKE-AfxuKw5bLtJC0MgoamDiyE-bcJNUJCXvtZX6A%40mail.gmail.com>.

1. GenomeSpecific
   1. HG001_het

regions containing putative compound heterozygous variants within 50bp of each other from v3.2.2 vcf of the GIAB samples HG001.

- 1. HG001_multi

regions containing multiple variants within 50bp of each other from v3.2.2 vcf of the GIAB samples HG001.

- 1. HG002_het

regions containing putative compound heterozygous variants within 50bp of each other from v3.2.2 vcf of the GIAB samples HG002.

- 1. HG002_multi

regions containing multiple variants within 50bp of each other from v3.2.2 vcf of the GIAB samples HG002.

1. LowComplexity
   1. TRBD

merged Repeats downloaded from TRDB for hg19 on 3/31/15.

- 1. TRBD_95

repeats with >=95% identity in Merged Repeats downloaded from TRDB for hg19 on 3/31/15.

- 1. TRBD_lt7_lt51_95…

subsetted repeats with >=95% identity in Merged Repeats downloaded from TRDB for hg19 on 3/31/15 into those with a unit length <=6bp or >6bp, and then to those with a total repeat length <50bp, 51-200bp, or >200bp. Regioin named as TRBD_{repeat unit}_{total repeat length}_{% identity}, which TRBD_gt6_lt101_95 contains repeats with a unit >6bp and a length <101bp with >95% identity.

- 1. SR_triTR_51to200…

Named as SimpleRepeat_{repeatunit}_{total repeat length}

- 1. AR_lt51_95

Simple homopolymers >5bp, simple tandem repeats 11-50bp, and all TRDB repeats <51bp

- 1. AR_51to200_95

Simple tandem repeats 51-200bp, and all TRDB repeats 51-200bp

1. Mappability

{length}_m{mismatch}_e{indel}

mappability files for 100 bp (l100), 125 bp (l125), 150 bp (l150), and 250 bp (l250) single-end reads, allowing:

a) 0 mismatches and 0 indels (m0_e0)

b) 1 mismatches and 0 indels (m1_e0)

c) 2 mismatches and 0 indels (m2_e0)

d) 2 mismatches and 1 indels (m2_e1)

1. SegmentalDuplications
   1. mm-2

created to identify regions of GRCh37 that have homology to the decoy sequence hs37d5

- 1. mm-2complement

the complement of mm-2

- 1. split

hg19_self_chain_split, in bedpe format to show pairs of sites with mapping homology

- 1. split_both

hg19_self_chain_split_both, a bed file with all sites in 3-column bed format

- 1. split_both_gt10k

hg19_self_chain_split_both_gt10k, a file with only regions >10kb in size

- 1. split_both_withalts_gt10k

created with regions>10kb with mapping homology to any other region of the genomes including ALT loci

**Table S1. Performances of variation calling under different filtering threshold conditions.**

| **Platform** | | **BGISEQ-500 PE50** | | | | **HiSeq2500 PE150** | | | |
| --- | --- | --- | --- | --- | --- | --- | --- | --- | --- |
| Low quality threshold^1^ | | Raw | 5 | 10 | 20 | Raw | 5 | 10 | 20 |
| #Raw read (Mb) | | 2712.21 | 2712.21 | 2712.21 | 2712.21 | 722.73 | 722.73 | 722.73 | 722.73 |
| #Raw base (Gb) | | 135.61 | 135.61 | 135.61 | 135.61 | 106.96 | 106.96 | 106.96 | 106.96 |
| #Clean read (Mb) | | - | 2517.91 | 2378.72 | 1986.15 | - | 714.322 | 665.27 | 616.31 |
| #Clean base (Gb) | | - | 125.89 | 118.93 | 99.30 | - | 105.720 | 98.46 | 91.21 |
| Clean rate | | - | 92.84% | 87.70% | 73.23% | - | 98.84% | 92.05% | 85.28% |
| Average depth(X) | | 42.19 | 41.97 | 39.65 | 33.10 | 34.34 | 35.24 | 32.82 | 30.40 |
| Mapping rate^2^ | | 97.03% | 97.40% | 97.87% | 98.01% | 95.13% | 95.39% | 95.74% | 95.89% |
| PE mapping rate | | 93.67% | 94.13% | 95.07% | 95.76% | 93.91% | 94.35% | 94.94% | 95.13% |
| SNP^2^ | Total | 3,432,752 | 3,451,992 | 3,451,124 | 3,456,593 | 3,590,726 | 3,471,918 | 3,621,362 | 3,585,132 |
|  | # TP | 3,003,491 | 3,009,130 | 3,006,132 | 3,009,670 | 3,091,530 | 3,069,135 | 3,101,286 | 3,080,815 |
|  | # FP | 12,812 | 14,990 | 15,203 | 16,525 | 5,836 | 3,952 | 6,028 | 6,321 |
|  | FPR | 0.00051% | 0.00059% | 0.00060% | 0.00065% | 0.00023% | 0.00016% | 0.00024% | 0.00025% |
|  | # FN | 189,466 | 183,828 | 186,825 | 183,287 | 101,428 | 123,823 | 91,672 | 112,142 |
|  | FNR | 5.93% | 5.76% | 5.85% | 5.74% | 3.18% | 3.88% | 2.87% | 3.51% |
|  | Sensitivity | 94.07% | 94.24% | 94.15% | 94.26% | 96.82% | 96.12% | 97.13% | 96.49% |
|  | PPV | 99.58% | 99.50% | 99.50% | 99.45% | 99.81% | 99.87% | 99.81% | 99.80% |
| INDEL^2^ | Total | 564,466 | 546,905 | 554,568 | 534,015 | 746,216 | 741,949 | 686,697 | 623,921 |
|  | # TP | 264,350 | 263,953 | 261,867 | 262,055 | 344,957 | 345,139 | 341,266 | 332,376 |
|  | # FP | 16,492 | 12,458 | 16,931 | 13,485 | 10,012 | 9,944 | 11,509 | 13,164 |
|  | FPR | 0.00065% | 0.00049% | 0.00067% | 0.00053% | 0.00040% | 0.00039% | 0.00046% | 0.00052% |
|  | # FN | 104,825 | 105222 | 107,311 | 107124 | 24,244 | 24063 | 27,940 | 36830 |
|  | FNR | 28.39% | 28.50% | 29.07% | 29.02% | 6.57% | 6.52% | 7.57% | 9.98% |
|  | Sensitivity | 71.61% | 71.50% | 70.93% | 70.98% | 93.43% | 93.48% | 92.43% | 90.02% |
|  | PPV | 94.13% | 95.49% | 93.93% | 95.11% | 97.18% | 97.20% | 96.74% | 96.19% |

Note:

1. Raw means raw data without filtering. Then we filtered the reads with more than 10% bases to be of low quality (with quality score lower than 5, 10 and 20 accordingly).
2. For this comparison, we used the same pipeline to do the variation calling (BWA aln for mapping and GATK UnifiedGenotyper for variation calling).
