# Supplementary material for: A reference human genome dataset of the BGISEQ-500 sequencer
Source: Gigascience. 2017 Apr 1;6(5):1–9. doi: 10.1093/gigascience/gix024 (PMC5467036; doi:10.1093/gigascience/gix024)
Supplement: Response_to_reviewer_comments_Original_Submission.pdf [file gix024_Response_to_reviewer_comments_Original_Submission.pdf]

To the Editor and Reviewers:
We would like to thank the editor and reviewers for careful reading, and constructive suggestions for our manuscript. According to comments from both reviewers, we have comprehensively revised our manuscript. We think we have addressed all the questions mentioned by the reviewers thus we are submitting our revised manuscript for your comments. In the meantime, we have obtained the pair-end 100 bp (PE100) data during the time of revision, thus we have included the PE100 data description and analysis in the revised manuscript. As a datanote which aims at publishing the dataset with basic description and assessment, we think after the revision, it is a more comprehensive representative dataset for the BGISEQ-500 sequencing platform. We are looking forward to your feedbacks and further comments/suggestions are welcome.

Below, we included the point-to-point response to the comments of both reviewers.

Reviewer #1: The authors present a useful public dataset from a new sequencing instrument. As far as I know, this is the first public dataset from the BGISEQ-500, so it's very useful to make this available. I recommend publication if more details about the methods are given, such as those below:

1. Please give version of all tools and exact parameters used in analysis pipeline. Was GATK unifiedgenotyper or haplotypecaller used?
Response:
We have revised the manuscript according the reviewers' comments with methods described in details, and furthermore, we have added the supplementary information with parameters indicated (Supplementary material).

2. What version of high-confidence calls from GIAB was used? The authors may want to use the most recent version available at ftp://ftp-trace.ncbi.nlm.nih.gov/giab/ftp/release/NA12878_HG001/latest/
Response:
We used Version 3.2 of the high-confidence calls in the previous manuscript, thus thanks to the suggestion of the reviewer, we have changed to version (Version 3.3) and updated all the statistics.

3. Would it be possible to stratify the false positives and negatives by genome context to better understand the strengths and weaknesses? For example, the authors could use bed files at https://github.com/ga4gh/benchmarking-tools/tree/master/resources/stratification-bed-files
Response:
Per this constructive suggestion, we have further assessed the false positive rate, false negative rate and sensitivity in different regions of the genome. Using the genome context file indicated by the reviewer, we assessed the false positive rate (FPR), false negative rate (FNR) and sensitivity in different regions and compared the BGISEQ-500 and Hiseq results. We have modified the manuscript accordingly to give this information (Supplementary material Figure S1). Especially, for the regions which were difficult for short reads mapping, we found better performance for the HiSeq2500 data than the BGISEQ-500 data, indicating discrepancy caused by shorter read length. And for the coding sequences, BGISEQ-500 had

performance similar to HiSeq2500 data reflecting feasibility of the new sequencing platform.

4. Could the authors label the Venn diagrams in the figure with the originating callsets to make it easier to interpret?
Response:
We have modified this figure accordingly. Also, we would like to point out that for variation calling parameters (FNR, FPR and sensitivity) estimation, we used the methods provided by GIAB instead of directly calculation from the Venn diagrams.

Reviewer #2: Review for "A reference human genome dataset of the BGISEQ-500 sequencer"

In this manuscript entitled "A reference human genome dataset of the BGISEQ-500 sequencer" Huang et al. present a sequencing dataset from BGI's recently released sequencing instrument BGISEQ-500. The authors have sequenced the Genome in a Bottle Consortium cell line (NA12878) using this new instrument and, in essence, performed some basic analysis and compared it's performance to previously data generated on Illumina's HiSeq2500. As this is the first time a BGISEQ-500 dataset is made available this manuscript will be of great value for the scientific community with interest to develop and adapt bioinformatics solutions to the BGISEQ-500, at least or specifically for researches in China (as of the current time the BGISEQ is limited to the Chinese market). Overall, this manuscript is viable and clearly structured. Nevertheless, I have some major and several minor concerns which should be addressed by the authors.
Response:
We would like to thank the reviewer for the positive comments. In addition to the previous data of PE50, we also included the PE100 data after the revision. More importantly, we have made revisions according to the reviewers' comments, and addressed all the concerns. We have listed our point-to-point responses to your questions below and revised the manuscript accordingly.

Major compulsory revisions:

Data availability: Albeit the authors have specified two repositories (the GigaDB repository with the URL ftp://user14@ and the ENA project identifier PRJEB15427) it was not possible for me to access the data, as the GigaDB access was either bound to the user "user14" for which the login credentials were missing (and the corresponding FTP directory was not accessible anonymously) or the ENA project identifier could not be found on the ENA websites (because the project was not activated yet?). Anyhow, it was not possible for me to to gain data access and therefore I could not verify fundamental claims in this manuscript or try to reproduce the results.
Response:
We had already released the access permission for public on ENA website(http://www.ebi.ac.uk/ena/data/view/PRJEB15427), and you also can access data on GigaDB (http://gigadb.org/dataset/100252). Also, we have uploaded the PE100 data which can also be found there now.

Sequence data summary; page 6 line 2ff: In this paragraph the authors describe how the data was prepared prior to the SNP analysis. The pre-processing of the data was kept very simple (which is not detrimental per se), i.e. filtering out any read which had more than 5 bases with a q-score below 10 or more than one ambiguous base call. Filtering on quality scores is one of the most basic and most applied pre-processing steps when dealing with NGS data, but is always dependent on the thresholds applied. For data generated by well known and established instruments, these thresholds emerged during various studies and user experience (to more or less a common practice). For new instruments it is therefore important to learn about the data quality in order to aid the decisions which thresholds give the most reasonable results and even if they should be applied at all. Of course, this is a dataset submission and not a comprehensive comparison study between the BGISEQ-500 and other instruments. Still, more questions emerged after reading this paragraph than could be answered. Did the authors have tried different filtering filtering methods and what were the results?

Response:

As mentioned by this reviewer, it is a dataset submission instead of a comprehensive comparison study, thus we are describing basic information of the dataset we submitted, in which we have also included some comparisons to reflect the data quality. We have included the detailed descriptions of software/pipelines with parameters, to justify all the comparisons.

For the data filtering, we used the filtering parameters which we commonly used for analyzing different datasets. So we didn't analyze how different filtering parameters might affect the results. According to the suggestion of this reviewer, we further analyzed whether the parameters would have substantially affected the variation calling and assessments.

We filtered reads with high proportion of low quality bases, we tested the results by setting three thresholds of low quality bases (quality score lower than 5, 10 and 20 respectively) (Rebuttal Table 1, see the supplementary materials). We found limited effects on the SNP results with the different parameters, while for indels, less stringent parameters would result in higher sensitivity and lower FNR. But the overall trend of the comparison was just the same. Although we didn't test all the combinations of the parameters, we anticipated the overall assessment to be not affected substantially thus we just showed the results based on the previous set of parameters.

How many reads were filtered due to inferior read quality or due to ambiguous bases (just provide the numbers)?

Response:

For the BGISEQ-500 PE100 data, 11.9% (9.2% low quality reads and 2.7% ambiguous reads) low quality reads were found and for PE50 data, 12.3% low quality raw reads were found (5.4% low quality reads and 6.9% ambiguous reads). For the HiSeq2500 data, for the consistency, we applied the same filtering parameter, and we identified 7.95% low quality reads (7.7% low quality reads and 0.25% ambiguous reads). We have indicated this in the revised manuscript (Page 6, Line 20 and Line 27).

Also, the authors state that FastQC was used "to conduct quality control" but it is not explained how that was actually done. FastQC itself just performs some basic statistics and test and reports on these mainly via appropriate plots. Which statistics were considered for quality control, what were the results of these and how was that interpreted and used in terms of quality controlling the data?

Response:
We used the parameters (10% bases with sequencing quality lower than 10, and more than 1% ambiguous bases) described for filtering and FastQC only to calculate basic statistics of the sequencing data (especially base quality distribution showed in Figure 4). Thus we did not use FastQC for data filtering.

In addition, when using FastQC's quality score distribution plots I would suggest to at least report also about the difference between raw and cleaned reads (the q-score distribution of the raw reads is considered of higher importance than those of pre-processed reads) and - this would be highly interesting - difference in the variant calling for raw and filtered data. Finally, why the exact same filtering was applied to the Illumina HiSeq2500 data?
Response:
We agreed with the reviewer that the base quality distribution of the raw reads is more important than that of the filtered reads. Thus we have modified in our revised manuscript to show the quality distributions of the raw data instead of the clean data in the revised Figure 4. Also, we are including a comparison of the quality distributions of both the raw data and the filtered data (Rebuttal Figure 1, see the supplementary materials).
From Rebuttal Figure 1, we found that the raw and filtered data have similar trends of base-wise quality score distribution with the minimum Q-score of each position read increased after filtering. Raw and filtered data of the two platforms had almost the same pattern of Q-score score distribution, but we found BGISEQ-500 q-score peaks to be at phred score of 35 and HiSeq2500's peak to be at score of 37, which means HiSeq2500 has higher quality proportions than BGISEQ-500.

Filtering on 10% q10 is usually not done on Illumina data (q20 is considered more appropriate), but it depends on the variant calling pipeline which filter thresholds may improve the results, if at all (if e.g. recalibration is applied filtering is not necessary, se also doi:10.1186/1471-2164-13-S8-S8 or doi:10.1038/srep17875). In order to allow the community to better understand and classify this data, results on different approaches would be highly appreciated (at least raw and cleaned).
Response:
We agree with the reviewer that filtering criteria for different sequencing platform should be different. As the description of the sequencing data, we used just one set of parameters to do the filtering instead of exploring the best combination. And also, we are now providing the raw data for the community to further analyze the dataset. During the revision, we have also conducted analysis using the raw data according to the suggestion of this reviewer (Rebuttal Table 2, see the supplementary materials). We found similar results for the raw data and clean data. Thus in the revision, we still provided the all the statistics based on the clean data.

Minor revisions:

English spelling and writing: There are several minor misspellings and formatting issues throughout the manuscript. I will list some examples below, but this list does not claim to be complete. I recommend to extensively prove read this paper in order to generally improve the writing.

- p2/l5: which can help to fulfill
- p2/l11: and compared it to
- p3/l7: technologies [2]. Thus it has been
- p4/l2: to DNA fragments between 50bp and 800bp
- p5/l15: 'After each correction step ' or 'After all correction steps '
- p6/l4: for each paired read,
- p6/l14: the Figure reference should be 4b and not 4c
- p7/l13: compared to the identified
- p8/l3: better written as: with sequencing length of 50bp for both paired reads
- p8/l7: the word "therefore" should be removed
- p8/l9: further reflecting
- p8/l10: "In the meantime" does make no sense here
- p8/l19: the further technical improvement and development

Response:

Thanks to the suggestion of the reviewer, we have revised accordingly.

Abstract; page 2 line 5: As a generalized statement, it is not clear how a new sequencer which, in terms of throughput and cost effectiveness, is comparable to existing solutions "can help [to] fulfill the growing demands for sequencing". This could be assumed to be true of this precise new instrument is superior in one or several key aspects, but this could not be shown in the course of this manuscript or was missed to be explained.

Response:

We thought even just alternative choice for large scale sequencing with good data quality would provide opportunities for more sequencing based studies and applications. So we used to mention it in the abstract. But we agreed to the reviewer that since we did not provide other information, we should not make this kind of statement thus we deleted it during the revision. Also for the other parts, we tried our best to avoid making this kind of judgement and just provide data and basic description during the revision.

Abstract; page 2 line 15; other locations: When comparing the data sets generated by the BGISEQ to a data set generated by HiSeq2500 platform it should be stated as such and not hidden behind the sentence 'other sequencing platforms' which erroneously indicate a comparison to different (at least two) platforms (which was not done in this article). The same holds true for related sentences in the results and discussion section (e.g. p3/l19, p8/l8, ...).

Response:

Thanks to the suggestion of the reviewer, we have changed the description of ‘other sequencing platform’ to avoid misleading.

Abstract; page 2 line 16ff: An abstract should be short but still precise. A 'relatively lower false positive rate and sensitivity' was not the result of this study, at least it was not shown. In the results section (and also in Figure 4b and 4c) an inferior sensitivity and FPR for SNPs and a remarkable lower sensitivity and FPR for indels was observed. In this manner, the identified higher error rates for the BGISEQ data were

not just "some discrepancies". This should be described more precisely.
Response:
Following this comment and the suggestions before, we have revised the abstract accordingly (revised manuscript P2, L13-19).

Sequence library preparation; page 4 line 1ff: This whole paragraph could be a bit more in detail describing how the complete library preparation was carried out and what were the individual conditions. Was the BGI's Sample Prep System used?
What was the configuration of the 8-cycle PCR, what the condition of the "special molecule" and the configuration of the splint circularization?
Response:
We have further modified the paragraph of library construction to make it clearer (revised manuscript P4, L12-28). Basically, we followed the manufacturer's instruction for library construction, using BGISP-100 (BGI sample preparation machine provided along with the sequencer).

Base calling and raw images; page 5 line 11ff: It would be interesting to learn more about the actual base calling process and how correction of e.g. cross-talk between different channels is achieved. The authors state in page four line 28ff, that a 'detailed description' on the base calling is given in this section. However, this description is at best a very brief overview just listing the three main steps of the base calling pipeline without any further explanation. In addition, it would also be of great value for the community to learn about how phred like quality scores were actually inferred from the base calling process.
Response:
We added more detailed cross-talk about base calling process and detailed phred score calculation accordingly (revised manuscript, P5-P6).

Variation calling; page 6 line 16ff: What for adjustments have been made to the GATK best practice pipeline and why? Why bwa-mem was not used on the Illumina data?
Response:
Considering about the efficiency of data analysis, we used SOAP gaea, which is a based on the Hadoop MapReduce parallel computing framework for whole genome resequencing analysis pipeline. Both bwa and gatk were re-written in the MapReduce framework. But during the revision, for the PE100 BGISEQ-500 and HiSeq2500 data, we used the GATK best practice pipeline (bwa-mem, haplotype caller) for the variation calling.
For the previous PE50 BGISEQ-500 data, so we didn't use bwa-mem, which is designed for longer sequences ranged from 70bp to 1Mbp. Moreover, we thought it would be better to use the same parameters for comparing the two platforms. But we also followed the reviewer's advice to compare bwa-mem and bwa-aln mapping and variants calling results (Rebuttal Table 3 and Rebuttal Table 4). We could find that bwa-mem does increase mapping rate for longer read, but the mismatch rate is also increased. For SNP and indel evaluations, we can find the results that using the two alignment algorithms are similar.

The information given on the tools and parameters used in Figure 4a would not be sufficient to reproduce the results (a list of all precise commands used could be provided e.g. in a supplement). Also, the information about which tool and their corresponding version is missing.
Response:
Sorry for missing the information, and we have added these details of all the parameters in the supplementary methods section (Supplement materials).

Variation calling; page 7 line 14ff: When speculating about the lower sensitivity of the BGISEQ data in the high confident region, could similar effects (dropping coverage) also be identified for the Illumina data? If so, did the authors tried to use a higher subset of the GIAB Illumina data and verified their hypotheses? What are the coverage ranges for the missed SNPs in this region (which is the desired base line coverage)? What is the coverage for the erroneously called SNPs (FPs)?
Response:
Following the suggestion of this reviewer, we have plotted the sequencing depth distribution of FP comparing to TP for both the BGISEQ-500 and HiSeq2500 data. Since we previously described only the PE50 data of BGISEQ-500, we are comparing the distributions of PE50 and HiSeq2500 in Rebuttale Figure 2. From this comparison, we can find a secondary depth peak in the distribution of FP from the BGISEQ-500 PE50 data while not in the FP from HiSeq2500 data. Thus further increasing sequencing depth of the BGISEQ-500 PE50 data might be able to reduce those FP with low sequencing depth.

Also, a more than doubled FPR (0.38% compared to 0.14%) cannot considered to be "similar". Finally, a clear inferior TPR on the indels of course shows a discrepancy of the dataset to identify indels, but what are the implications on the BGISEQ (e.g. homopolymer errors)?
Response:
According to the comments of both reviewers, we have changed to use version 3.3 of high-confidence variants sets for the assessment and comparison. The FPR of BGISEQ-500 PE50 is 0.00088%, and the FPR of BGISEQ-500 PE100 data is 0.00067%, comparing to 0.00046% of the HiSeq2500 PE150 data.

Discussion; page 8 line 6ff: A single base quality is - to my understanding - not the mapping rate / mapping quality.
Response:
Sorry for the misleading. We would like to explain that sequencing quality (single base quality and read quality) will affect analysis metrics (mapping rate and unique mapping rate, etc.). We have modified this sentence to avoid misleading.

Discussion; page 8 line 11ff: How will a longer sequencing length improve the indel identification?
Response:
Shorter reads would be result in more difficulty and errors for the mapping, thus the detection of indels with shorter sequencing length would be more difficult. As described previously [http://bib.oxfordjournals.org/content/14/1/46.full] (see Figure 2 of this paper), raising read length significantly increase sensitivity and decrease false negative rate (FNR) of indels detection. In the meantime, it can also be reflected from comparison between the PE50 and PE100 data of BGISEQ-500,

which other sequencing quality and features should be similar.

Figures & Legends

Figure1: I would recommend to separate both figure parts in individual ones, as the whole figure is a bit disorganized.
Response:
We had adjusted composing of Figure 1 accordingly.

Figure2: The fourth sentence ("In order to correct ...") seems to be incomplete (after "and chemical").
Response:
Sorry for misleading, we had already edited the sentence.

Figure 3: It would be helpful if the plots for the forward and reverse reads were separated visually either buy appropriate sub-plotting or by using distinct visual separators. The legend of this figure should be rewritten as a whole. Sub-plots do not necessarily need individual sub-headings, they can be indicated inline (as done in Figure1), which would remove redundant text. Also, interpretation of results are usually not part of a figure legend but part of the corresponding manuscript text (lower quality, similar distribution).
Response:
We had adjusted composing of Figure 3 legend accordingly.

Figure4: Comma is missing after "filtering, alignment,". In the picture "false positive" should be replaced with "false positive rate" or "FPR" (with an appropriate legend text).
Response:
Thanks for your suggestion. After the revision, we switched to use rtg-tools software to evaluate the performances of variation calling, so we removed all the Venn diagrams. Instead of this, we added Table 4 to show detailed variants evaluation results in the revised manuscript.

Tables

Table1: How was the error rate calculated (based on mapping?)? This information is completely missing in the manuscript.
Response:
Error rate was calculated by this formula: , which Q is Phred quality scores, we calculate P of each base and then average them. Since we simply calculated the average error rate of all the bases, we thought it would be inappropriate thus we have deleted this column in the revised manuscript.

Table3: In the legend there are abbreviations explained (TP, FP,) which are not given in the table. Is the table missing this information or is the legend wrong?
Response:
We had a previous version of the table that showed TP, FP, etc. metrics. Since we showed these results

in Figure 4, we deleted those rows but forgot to delete these explanations. We have deleted them during revision.

In addition, when also considering Figure4 (where this description would be more appropriated) TP and TN are interchangeable (but authors definition they are the same), which should be stated as such.
Response:
As mentioned earlier in respond to previous comments, now we switched to use rtg-tools software to evaluate the performances of variation calling, so we removed all the Venn diagrams. Instead of this, we added Table 4 to show detailed variants evaluation results in revised manuscript.
