# Supplementary material for: A reference human genome dataset of the BGISEQ-500 sequencer
Source: Gigascience. 2017 Apr 1;6(5):1–9. doi: 10.1093/gigascience/gix024 (PMC5467036; doi:10.1093/gigascience/gix024)
Supplement: Response_to_reviewer_comments_Revision_1.pdf [file gix024_Response_to_reviewer_comments_Revision_1.pdf]

To the Editor and Reviewer:
We would like to thank the editor and reviewer for acceptance for our previous revisions and further suggestions. We have further revised our manuscript according to comments of reviewer #2. Hopefully, it resolves all the questions raised and meets the requirement for publication.

Following are our responses to the comments for reviewer #2
Reviewer #2: Following up on the revised manuscript presented by the authors I appreciate the effort that was put into addressing the issues raised previously. Even though, the previous submission constituted an interesting and useful dataset for the new BGISEQ-500 sequencing instrument, the resubmission now includes not only all the information necessary to reproduce the presented dataset and results but overall is also more clear with respect to the library preparation and sequencing, the effect of the different processing steps, including their parameters, and the conclusion which were drawn upon them. In particular, I appreciate that the authors could show via the Rebuttal, to sufficient extent, that the main comparison is not (heavily) biased by the choice of the processing tools used as well as the filtering and mapping parameters, which in turn adds much to the robustness of the presented manuscript.
Response:
Thanks for accepting our revision. And we further revised the manuscript according to this reviewer's suggestions as below.

1) Regarding Rebuttal Table 1 and 2, it is interesting to see that variant efficiency in terms of FPR and FNR for the indel SNPs is slightly better for less stringent parameters and for the raw than data sets than for the high stringent and cleaned ones. Albeit these differences are very small, as they are for all other metrics, the fact that data cleaning had such a subtle effect in general might also be interesting for the community. Therefore, the authors could include these finding additionally in the supplement material.
Response:
Thanks for the suggestion and we have included this information in Table S1. We also have revised the manuscript to describe the analysis (Page 6 line20-22) as follows.
"Although previous studies revealed that raw data filtering would not substantially affect variation calling result [7, 8], we found slightly different performances of variation calling using different raw data filtering criteria (Table S1)."

2) Page 4 line 24: "we referred" seem to be wrong in time, either "we refer" or "we followed" should be more correct.
Response:
We had revised this accordingly (changed to "we followed" as suggested by the reviewer).

3) I found the added details on base calling and cross-talk correction interesting and in particular helpful to better understand how this important step is carried out on the BGISEQ. However, the middle part of this subsection could take some minor adjustments to aid comprehensibility, e.g.:
- page 5 line 19: add comma to sub-clause "...of the four intensities and, to keep .. or noisy, we take"
- page 5 line 20: maybe replace "all non-maximum intensities" with something like "that have less than 80% of the C intensity for the remaining three other intensities"
Response:

Sorry for the above two confusing sentences and we had adjusted description accordingly.

- page 5 line 23: This sentence seems to miss a verb and therefore some meaningful sense.
Response:
Thanks for your corrections, we had revised this sentences accordingly (as follows).
“Then, the linear regression was carried out for these background G intensities as a function of the C intensities.”

4) Page 7 line 14: Either there is missing one example in this enumeration or the comma between "duplication rate, mismatch rate" should be replaced by an and. In Addition, this isn't really a higher mismatch rate (.56% vs .34% and .58%) - maybe this is the missing information?
Response:
We edited this sentence accordingly. To make it clear, we only compared BGISEQ-500 PE100 and HiSeq2500 PE150, thus conclusion is slight higher duplication rate and comparable mismatch rate (as follows).
“We also observed slightly higher duplication rate and comparable mismatch rate in the BGISEQ-500 PE100 dataset comparing to the HiSeq2500 data (Table 2)”

5) Page 7 line 17: Not less, but a very little more than 3.6 million SNPs were identified.
Response:
We changed ‘less’ to ‘more’ accordingly.

6) Page 8 line 1ff: The numbers presented here in parentheses doesn't match those ones given in Table 4.
Response:
Thanks for pointing out our mistakes, and we had changed it accordingly.

7) Page 8 line 14: I could not find supplementary TableS1 in the supplement.
Response:
This is also a typo, ‘table S1’ should be Figure S1, we had corrected it accordingly.

6) Page 9 line 10: If "etc." is at end of a sentence, no other period is added.
Response:
We had revised it accordingly.
