# Supplementary material for: A reference human genome dataset of the BGISEQ-500 sequencer
Source: Gigascience. 2017 Apr 1;6(5):1–9. doi: 10.1093/gigascience/gix024 (PMC5467036; doi:10.1093/gigascience/gix024)
Supplement: Reviewer_1_Report_(Original_Submission).pdf [file gix024_Reviewer_1_Report_(Original_Submission).pdf]

**Reviewer Report**

**Title:** "A reference human genome dataset of the BGISEQ-500 sequencer"

**Version:** Original Submission **Date:** 10/11/2016

**Reviewer name:** Justin Zook

**Reviewer Comments to Author:**

The authors present a useful public dataset from a new sequencing instrument. As far as I know, this is the first public dataset from the BGISEQ-500, so it's very useful to make this available. I recommend publication if more details about the methods are given, such as those below:

1. Please give version of all tools and exact parameters used in analysis pipeline. Was GATK unifiedgenotyper or haplotypecaller used?

2. What version of high-confidence calls from GIAB was used? The authors may want to use the most recent version available at ftp://ftp-trace.ncbi.nlm.nih.gov/giab/ftp/release/NA12878_HG001/latest/

3. Would it be possible to stratify the false positives and negatives by genome context to better understand the strengths and weaknesses? For example, the authors could use bed files at

https://github.com/ga4gh/benchmarking-tools/tree/master/resources/stratification-bed-files

4. Could the authors label the Venn diagrams in the figure with the originating callsets to make it easier to interpret?

**Level of Interest**

Please indicate how interesting you found the manuscript: An article of importance in its field

**Quality of Written English**

Please indicate the quality of language in the manuscript: Acceptable

**Declaration of Competing Interests**

Please complete a declaration of competing interests, considering the following questions:

- Have you in the past five years received reimbursements, fees, funding, or salary from an organisation that may in any way gain or lose financially from the publication of this manuscript, either now or in the future?
- Do you hold any stocks or shares in an organisation that may in any way gain or lose financially from the publication of this manuscript, either now or in the future?
- Do you hold or are you currently applying for any patents relating to the content of the manuscript?
- Have you received reimbursements, fees, funding, or salary from an organization that holds or has applied for patents relating to the content of the manuscript?
- Do you have any other financial competing interests?
- Do you have any non-financial competing interests in relation to this paper?

If you can answer no to all of the above, write 'I declare that I have no competing interests' below. If your reply is yes to any, please give details below.

I declare that I have no competing interests

I agree to the open peer review policy of the journal. I understand that my name will be included on my report to the authors and, if the manuscript is accepted for publication, my named report including any attachments I upload will be posted on the website along with the authors' responses. I agree for my report to be made available under an Open Access Creative Commons CC-BY license (http://creativecommons.org/licenses/by/4.0/). I understand that any comments which I do not wish to be included in my named report can be included as confidential comments to the editors, which will not be published.

I agree to the open peer review policy of the journal
