# Supplementary material for: A reference human genome dataset of the BGISEQ-500 sequencer
Source: Gigascience. 2017 Apr 1;6(5):1–9. doi: 10.1093/gigascience/gix024 (PMC5467036; doi:10.1093/gigascience/gix024)
Supplement: Reviewer_2_Report_(Original_Submission).pdf [file gix024_Reviewer_2_Report_(Original_Submission).pdf]

Reviewer Report

Title: "A reference human genome dataset of the BGISEQ-500 sequencer"

Version: Original Submission **Date:** 10/19/2016

Reviewer name: Sebastian Jünemann

Reviewer Comments to Author:

Review for "A reference human genome dataset of the BGISEQ-500 sequencer"

In this manuscript entitled "A reference human genome dataset of the BGISEQ-500 sequencer" Huang et al. present a sequencing dataset from BGI's recently released sequencing instrument BGISEQ-500. The authors have sequenced the Genome in a Bottle Consortium cell line (NA12878) using this new instrument and, in essence, performed some basic analysis and compared it's performance to previously data generated on Illumina's HiSeq2500. As this is the first time a BGISEQ-500 dataset is made available this manuscript will be of great value for the scientific community with interest to develop and adapt bioinformatics solutions to the BGISEQ-500, at least or specifically for researches in China (as of the current time the BGISEQ is limited to the Chinese market). Overall, this manuscript is viable and clearly structured. Nevertheless, I have some major and several minor concerns which should be addressed by the authors.

Major compulsory revisions:

Data availability: Albeit the authors have specified two repositories (the GigaDB repository with the URL ftp://user14@ and the ENA project identifier PRJEB15427) it was not possible for me to access the data, as the GigaDB access was either bound to the user "user14" for which the login credentials were missing (and the corresponding FTP directory was not accessible anonymously) or the ENA project identifier could not be found on the ENA websites (because the project was not activated yet?). Anyhow, it was not possible for me to to gain data access and therefore I could not verify fundamental claims in this manuscript or try to reproduce the results.

Sequence data summary; page 6 line 2ff: In this paragraph the authors describe how the data was prepared prior to the SNP analysis. The pre-processing of the data was kept very simple (which is not detrimental per se), i.e. filtering out any read which had more than 5 bases with a q-score below 10 or more than one ambiguous base call. Filtering on quality scores is one of the most basic and most applied

pre-processing steps when dealing with NGS data, but is always dependent on the thresholds applied. For data generated by well known and established instruments, these thresholds emerged during various studies and user experience (to more or less a common practice). For new instruments it is therefore important to learn about the data quality in order to aid the decisions which thresholds give the most reasonable results and even if they should be applied at all. Of course, this is a dataset submission and not a comprehensive comparison study between the BGISEQ-500 and other instruments. Still, more questions emerged after reading this paragraph than could be answered. Did the authors have tried different filtering filtering methods and what were the results? How many reads were filtered due to inferior read quality or due to ambiguous bases (just provide the numbers)? Also, the authors state that FastQC was used "to conduct quality control" but it is not explained how that was actually done. FastQC itself just performs some basic statistics and test and reports on these mainly via appropriate plots. Which statistics were considered for quality control, what were the results of these and how was that interpreted and used in terms of quality controlling the data? In addition, when using FastQC's quality score distribution plots I would suggest to at least report also about the difference between raw and cleaned reads (the q-score distribution of the raw reads is considered of higher importance than those of pre-processed reads) and - this would be highly interesting - difference in the variant calling for raw and filtered data. Finally, why the exact same filtering was applied to the Illumina HiSeq2500 data? Filtering on 10% q10 is usually not done on Illumina data (q20 is considered more appropriate), but it depends on the variant calling pipeline which filter thresholds may improve the results, if at all (if e.g. recalibration is applied filtering is not necessary, se also doi:10.1186/1471-2164-13-S8-S8 or doi:10.1038/srep17875). In order to allow the community to better understand and classify this data, results on different approaches would be highly appreciated (at least raw and cleaned).

Minor revisions:

English spelling and writing: There are several minor misspellings and formatting issues throughout the manuscript. I will list some examples below, but this list does not claim to be complete. I recommend to extensively prove read this paper in order to generally improve the writing.

- p2/l5: … which can help to fulfill …

- p2/l11: … and compared it to …

- p3/l7: … technologies [2]. Thus it has been …

- p4/l2: … to DNA fragments between 50bp and 800bp

- p5/l15: 'After each correction step …' or 'After all correction steps …'

- p6/l4: … for each paired read, …

- p6/l14: the Figure reference should be 4b and not 4c

- p7/l13: … compared to the identified …

- p8/l3: better written as: … with sequencing length of 50bp for both paired reads …

- p8/l7: the word "therefore" should be removed

- p8/l9: … further reflecting …

- p8/l10: "In the meantime" does make no sense here

- p8/l19: … the further technical improvement and development …

Abstract; page 2 line 5: As a generalized statement, it is not clear how a new sequencer which, in terms of throughput and cost effectiveness, is comparable to existing solutions "can help [to] fulfill the growing demands for sequencing". This could be assumed to be true of this precise new instrument is superior in one or several key aspects, but this could not be shown in the course of this manuscript or was missed to be explained.

Abstract; page 2 line 15; other locations: When comparing the data sets generated by the BGISEQ to a data set generated by HiSeq2500 platform it should be stated as such and not hidden behind the sentence 'other sequencing platforms' which erroneously indicate a comparison to different (at least two) platforms (which was not done in this article). The same holds true for related sentences in the results and discussion section (e.g. p3/l19, p8/l8, ...).

Abstract; page 2 line 16ff: An abstract should be short but still precise. A 'relatively lower false positive rate and sensitivity' was not the result of this study, at least it was not shown. In the results section (and also in Figure 4b and 4c) an inferior sensitivity and FPR for SNPs and a remarkable lower sensitivity and FPR for indels was observed. In this manner, the identified higher error rates for the BGISEQ data were not just "some discrepancies". This should be described more precisely.

Sequence library preparation; page 4 line 1ff: This whole paragraph could be a bit more in detail describing how the complete library preparation was carried out and what were the individual conditions. Was the BGI's Sample Prep System used? What was the configuration of the 8-cycle PCR, what the condition of the "special molecule" and the configuration of the splint circularization?

Base calling and raw images; page 5 line 11ff: It would be interesting to learn more about the actual base calling process and how correction of e.g. cross-talk between different channels is achieved. The authors state in page four line 28ff, that a 'detailed description' on the base calling is given in this section. However, this description is at best a very brief overview just listing the three main steps of the base calling pipeline without any further explanation. In addition, it would also be of great value for the community to learn about how phred like quality scores were actually inferred from the base calling process.

Variation calling; page 6 line 16ff: What for adjustments have been made to the GATK best practice pipeline and why? Why bwa-mem was not used on the Illumina data? The information given on the tools and parameters used in Figure 4a would not be sufficient to reproduce the results (a list of all precise commands used could be provided e.g. in a supplement). Also, the information about which tool and their corresponding version is missing.

Variation calling; page 7 line 14ff: When speculating about the lower sensitivity of the BGISEQ data in the high confident region, could similar effects (dropping coverage) also be identified for the Illumina data? If so, did the authors tried to use a higher subset of the GIAB Illumina data and verified their hypotheses? What are the coverage ranges for the missed SNPs in this region (which is the desired base line coverage)? What is the coverage for the erroneously called SNPs (FPs)? Also, a more than doubled FPR (0.38% compared to 0.14%) cannot considered to be "similar". Finally, a clear inferior TPR on the indels of course shows a discrepancy of the dataset to identify indels, but what are the implications on the BGISEQ (e.g. homopolymer errors)?

Discussion; page 8 line 6ff: A single base quality is - to my understanding - not the mapping rate / mapping quality.

Discussion; page 8 line 11ff: How will a longer sequencing length improve the indel identification?

Figures & Legends

Figure1: I would recommend to separate both figure parts in individual ones, as the whole figure is a bit disorganized.

Figure2: The fourth sentence ("In order to correct ...") seems to be incomplete (after "and chemical").

Figure 3: It would be helpful if the plots for the forward and reverse reads were separated visually either buy appropriate sub-plotting or by using distinct visual separators. The legend of this figure should be rewritten as a whole. Sub-plots do not necessarily need individual sub-headings, they can be indicated inline (as done in Figure1), which would remove redundant text. Also, interpretation of results are usually not part of a figure legend but part of the corresponding manuscript text (lower quality, similar distribution).

Figure4: Comma is missing after "filtering, alignment,". In the picture "false positive" should be replaced with "false positive rate" or "FPR" (with an appropriate legend text).

Tables

Table1: How was the error rate calculated (based on mapping?)? This information is completely missing in the manuscript.

Table3: In the legend there are abbreviations explained (TP, FP, …) which are not given in the table. Is the table missing this information or is the legend wrong? In addition, when also considering Figure4 (where this description would be more appropriated) TP and TN are interchangeable (bu authors definition they are the same), which should be stated as such.

Level of Interest

Please indicate how interesting you found the manuscript: An article whose findings are important to those with closely related research interests

Quality of Written English

Please indicate the quality of language in the manuscript: Needs some language corrections before being published

Declaration of Competing Interests

Please complete a declaration of competing interests, considering the following questions:

- Have you in the past five years received reimbursements, fees, funding, or salary from an organisation that may in any way gain or lose financially from the publication of this manuscript, either now or in the future?
- Do you hold any stocks or shares in an organisation that may in any way gain or lose financially from the publication of this manuscript, either now or in the future?
- Do you hold or are you currently applying for any patents relating to the content of the manuscript?
- Have you received reimbursements, fees, funding, or salary from an organization that holds or has applied for patents relating to the content of the manuscript?
- Do you have any other financial competing interests?
- Do you have any non-financial competing interests in relation to this paper?

If you can answer no to all of the above, write 'I declare that I have no competing interests' below. If your reply is yes to any, please give details below.

I declare that I have no competing interests.

I agree to the open peer review policy of the journal. I understand that my name will be included on my report to the authors and, if the manuscript is accepted for publication, my named report including any attachments I upload will be posted on the website along with the authors' responses. I agree for my report to be made available under an Open Access Creative Commons CC-BY license (http://creativecommons.org/licenses/by/4.0/). I understand that any comments which I do not wish to be included in my named report can be included as confidential comments to the editors, which will not be published.

I agree to the open peer review policy of the journal
