# Supplementary material for: A reference human genome dataset of the BGISEQ-500 sequencer
Source: Gigascience. 2017 Apr 1;6(5):1–9. doi: 10.1093/gigascience/gix024 (PMC5467036; doi:10.1093/gigascience/gix024)
Supplement: Reviewer_2_Report_(Revision_1).pdf [file gix024_Reviewer_2_Report_(Revision_1).pdf]

Reviewer Report

Title: "A reference human genome dataset of the BGISEQ-500 sequencer"

Version: Revision 1 **Date:** 3/6/2017

Reviewer name: Sebastian Jünemann

Reviewer Comments to Author:

Following up on the revised manuscript presented by the authors I appreciate the effort that was put into addressing the issues raised previously. Even though, the previous submission constituted an interesting and useful dataset for the new BGISEQ-500 sequencing instrument, the resubmission now includes not only all the information necessary to reproduce the presented dataset and results but overall is also more clear with respect to the library preparation and sequencing, the effect of the different processing steps, including their parameters, and the conclusion which were drawn upon them. In particular, I appreciate that the authors could show via the Rebuttal, to sufficient extent, that the main comparison is not (heavily) biased by the choice of the processing tools used as well as the filtering and mapping parameters, which in turn adds much to the robustness of the presented manuscript.

In this manner, there are only minor issues left in the manuscript that, so my humble suggestion, could be addressed without the need for a further reviewing process.

1) Regarding Rebuttal Table 1 and 2, it is interesting to see that variant efficiency in terms of FPR and FNR for the indel SNPs is slightly better for less stringent parameters and for the raw than data sets than for the high stringent and cleaned ones. Albeit these differences are very small, as they are for all other metrics, the fact that data cleaning had such a subtle effect in general might also be interesting for the community. Therefore, the authors could include these finding additionally in the supplement material.

2) Page 4 line 24: "we referred" seem to be wrong in time, either "we refer" or "we followed" should be more correct.

3) I found the added details on base calling and cross-talk correction interesting and in particular helpful to better understand how this important step is carried out on the BGISEQ. However, the middle part of this subsection could take some minor adjustments to aid comprehensibility, e.g.:

- page 5 line 19: add comma to sub-clause "...of the four intensities and, to keep .. or noisy, we take"

- page 5 line 20: maybe replace "all non-maximum intensities" with something like "that have less than 80% of the C intensity for the remaining three other intensities"

- page 5 line 23: This sentence seems to miss a verb and therefore some meaningful sense.

4) Page 7 line 14: Either there is missing one example in this enumeration or the comma between "duplication rate, mismatch rate" should be replaced by an and. In Addition, this isn't really a higher mismatch rate (.56% vs .34% and .58%) - maybe this is the missing information?

5) Page 7 line 17: Not less, but a very little more than 3.6 million SNPs were identified.

6) Page 8 line 1ff: The numbers presented here in parentheses doesn't match those ones given in Table 4.

7) Page 8 line 14: I could not find supplementary TableS1 in the supplement.

6) Page 9 line 10: If "etc." is at end of a sentence, no other period is added.

Level of Interest

Please indicate how interesting you found the manuscript: An article of importance in its field

Quality of Written English

Please indicate the quality of language in the manuscript: Acceptable

Declaration of Competing Interests

Please complete a declaration of competing interests, considering the following questions:

- Have you in the past five years received reimbursements, fees, funding, or salary from an organisation that may in any way gain or lose financially from the publication of this manuscript, either now or in the future?
- Do you hold any stocks or shares in an organisation that may in any way gain or lose financially from the publication of this manuscript, either now or in the future?

- Do you hold or are you currently applying for any patents relating to the content of the manuscript?
- Have you received reimbursements, fees, funding, or salary from an organization that holds or has applied for patents relating to the content of the manuscript?
- Do you have any other financial competing interests?
- Do you have any non-financial competing interests in relation to this paper?

If you can answer no to all of the above, write 'I declare that I have no competing interests' below. If your reply is yes to any, please give details below.

I declare that I have no competing interests.

I agree to the open peer review policy of the journal. I understand that my name will be included on my report to the authors and, if the manuscript is accepted for publication, my named report including any attachments I upload will be posted on the website along with the authors' responses. I agree for my report to be made available under an Open Access Creative Commons CC-BY license (http://creativecommons.org/licenses/by/4.0/). I understand that any comments which I do not wish to be included in my named report can be included as confidential comments to the editors, which will not be published.

I agree to the open peer review policy of the journal
